# Clinical Review, and Hospital Reports

**Published:** 1838-04-01

**Authors:** 


### Clinical Review.

### Report of the Kent and Canterbury Hospital.

### Fever from Prolonged Lactation.—(*Continued.*)

The symptoms attendant upon the incipient, or latent, stage of this disease are the same as those described when treating of chlorotic fever; and are, in fact, common to the undeveloped stage of all fevers whatsoever. They are such as result from an enfeebled state of the system (however produced), and from a consequently imperfect performance of the several functions.

When the disease is fully developed, the following set of symptoms present themselves.

Evident loss of flesh; great prostration of strength; depression of spirits; frequent chills, particularly towards evening; countenance pale; eyes heavy, and sight occasionally dim; head giddy and painful, (sometimes vertex and occiput are the seat of pain, though it more frequently affects the left temple); respiration hurried; heart irregular and tumultuous in its action; pain in epigastrium, aggravated generally after food, and accompanied with a sense of sinking, and extreme faintness; appetite impaired; tongue thinly furred; bowels costive; pulse seldom under 90.

In such a state of the system, hysteria seldom fails to show itself, particularly if the patient is constitutionally predisposed to its attacks.

The foregoing symptoms bear, throughout, a striking general resemblance to those of chlorotic fever; to which, indeed, this disease is in its nature very closely allied. The skin, however, is seldom so much altered from its natural appearance; and there is, I have remarked, more epigastric pain and weakness than in this latter complaint.

The period of lactation at which the symptoms may first shew themselves, is very various. I have seen them occur at all periods, from the third to the sixteenth month. In proportion as the patients are naturally feeble of constitution, and ill-fed, does the disease display itself early. It is, as might be expected, most common in the lowest rank of life. But delicate mothers, in all grades of society, are liable to it.

The catamenia sometimes appear during its existence; but this is by no means what ordinarily happens. Generally there is more or less leucorrhœal discharge.

The pain in the *left* temple is sometimes so severe as to throw into the background (with the patient at least) all the other symptoms. It is described as a concentrated agonizing sensation with a feeling as if there were some violent pressure from within. I have met with a very few cases in which the *right* temple was the one thus affected.

In this, as in most of the other diseases of females attended with debility, there is generally pain in the left side, and (what is often mentioned as such) pain in the left iliac region; the latter produced, no doubt, by flatulent distention of the sigmoid flexure of the colon. It proves occasionally a very troublesome symptom.

Another very common seat of pain is the right hypochondriac region, from which in general the pain shoots backwards to the inferior angle of the scapula. But this is nothing more than a symptom of disordered digestion, as carried on in the upper part of the intestinal canal, (I dare almost venture to specify the duodenum) and is mentioned only as constituting a frequent complication in the disease of which we here treat.

When the disease attacks a mother in whose constitution the germs of tubercles exist, there is great danger of their rapid development. In such a case, it often occurs at a comparatively early period after the commencement of lactation.

Baillou noticed this disease; and, after him, Ramazzini, who mentions some of its leading symptoms. Dr. Marshall Hall has described it accurately. I had been aware of its existence, and had written a description of it, long before I was acquainted with the writings of these authors. The present description is given solely from my own observation. In saying this, however, I would not be understood as presuming on my own accuracy. The main object of these pages is to record the results of my individual experience, not to compile the statements and opinions of others. This would be only to add to the dead weight which already oppresses medical literature. A very few atoms of knowledge, *added to each other, times without number*, have produced the greater part of that enormous mass of bibliography under which our science at present groans and is stifled.

But this digression leads me to another, on a subject equally correlative to the main design of my Report. I mean the introduction of illustrative and confirmatory inferences, derived from my general experience, whether in public or in private practice. To confine my remarks strictly to those cases the details of which are given, would render the task in which I am engaged an exceedingly uninteresting and profitless one. The great end of science is to arrive at a knowledge of ultimate general laws; and he who would make any progress towards its attainment, must extend the field of his observations as widely as possible. He must see objects, however, with his own eyes, and not with the eyes of another; for without a previous knowledge of some common principle by which to determine their suitable collocation, no power of digesting, arranging, and condensing the observations of other men, can enable the mind to take at once a comprehensive and an accurate survey of them. A general law may be *proved* by the observations of others; it must be *discovered* by one's own.

But to return to the disease in question. Having stated my opinion of its *general* nature, and described its essential and complicating symptoms, I come now to consider its treatment. This may be very briefly discussed. Pure air, mild aperients, gentle tonics, blisters applied to the epigastrium, or, if need be, to the præcordial region—these will be found sufficient for the cure of the disease, *provided the child be at once and altogether* weaned.* Without this, all remedies must prove unavailing.

It may seem scarcely necessary to prohibit venesection in this disease, yet I have more than once seen it practised; the severe palpitation, accompanied with a hysterical sense of suffocation, misleading the practitioner into a belief that thoracic inflammation existed. Even leeching, I think, had better be avoided, blisters always answering the purpose better. This remark holds good with regard to all atonic congestions whatsoever, in which blisters not only relieve vascular fulness, but stimulate also to a healthier action.

When the pain in the left temple is very distressing, the external application of half a grain of morphia scarcely ever fails to relieve it. In a few cases I have known it to occur periodically, and then the exhibition of five grains of quinine three times daily, speedily removed it. In other cases, an emetic has proved highly serviceable, preventing altogether its periodical recurrence. I mention these facts chiefly with a view to shew that I by no means recommend an unvarying line of treatment. To do so with reference to any particular

---

* Mothers in the lower ranks of life, when ordered to wean their children, often do so only *in part*. They think that by continuing to nurse, they shall prevent another conception. This notion is ridiculed by modern obstetrical writers; but my own observation would lead me to believe that it has some foundation in truth. Yet in both the cases which I am about to detail, menstruation had returned, although the patients were still giving suck.

disease would be foolish enough; as regards a mere symptom, it would be the very height of absurdity.

I shall now proceed to detail briefly two of the cases. The third patient not attending regularly was discharged soon after her admission.

A. B., æt. 28, admitted an out-patient August 26th.

*Symptoms.*—Pain in occiput, also in back and loins; respiration hurried; impulse and sound of heart increased; ankles swelled towards evening; bowels slow; catamenia during last three months regular but scanty. Is suckling a child 15 months old.

She was ordered to wean the child, and had prescribed for her some mild aperient pills, together with a mixture containing ammonia. Under this simple treatment she recovered rapidly, and was discharged, cured, on the 9th of September.

The other case was much more tedious, and I had good reason to believe that the patient did not wholly wean her child until a late period of her attendance. In the very outset, she shewed great reluctance to the step, and assigned as a reason, the notion stated in note in the previous page.

The case was as follows:

H. P., æt. 28, admitted an out-patient, September 16th.

*Symptoms.*—Great general debility; countenance pale and distressed; palpitation of heart; pain in epigastrium and lower part of the sternum, extending backwards to between scapula; rigors every afternoon between 5 and 6 o'clock; pulse 90, feeble; tongue clean; bowels regular.

Is suckling a child, twelve months old, and menstruated last week.

*Treatment.*—Blisters to the epigastrium, mild aperients, a light bitter infusion, and, at a later period, quinine in five-grain doses.

Under the use of these remedies the symptoms seemed slightly to abate; but the patient did not gather strength, and there were soon added to the foregoing symptoms, heavy dragging pains in lower part of the abdomen, globus, hysterical swoonings, &c.; all of which, in a more or less severe form, continued until the middle of December, when, without any material change in the treatment, the patient began to recover, and in little more than a fortnight was completely restored to health. I have no doubt that up to the period when the improvement commenced, she had still continued to nurse her child.

### Gastro-Enteric Fever.

I use this as a specific term, including two varieties, namely *gastro-enteritis,* and *mucous fever;* the former of which presents itself in a *continued,* the latter in a *remittent* form. I believe that the difference between them arises from the existence in the former of inflammation, accompanied, or not, with ulceration of the mucous follicles—constituting the *dothinterite* of Bretonneau, the *exantheme intestinal* of Andral and other French authors. By the majority of French pathologists these two distinct types of fever are confounded, whence has originated serious error, both in doctrine and in practice; while none of the writers who have controverted their views, have (as far as I am aware) drawn the line of distinction so clearly as they might have done. That the distinction here insisted on, exists in nature, cannot be doubted; and it must be regarded more than it has hitherto been, if we would have less confused descriptions, and less (apparently) discordant plans of treatment.

*Gastro-enteric fever* is something more than simple fever, complicated in its progress with gastro-enteric derangement. It is fever presenting *at its very outset* (that is in its first *visible* stage) disorder of the stomach and bowels much graver than is ever witnessed in simple fever. The nervous system consequently sympathizes more strongly than in this latter, and the whole disease is, as every experienced practitioner knows, much more serious in its nature.

These then are the points of difference on which I would ground my distinction—the earlier occurrence,* and graver nature of the gastro-enteric disorder, and the more marked sympathy of the nervous system, particularly over that part of it which presides over the functions of relation.

I speak here more particularly of the *continued* form of gastro-enteric fever, that to which I have assigned the name *gastro-enteritis,* which is always attended with inflammatory symptoms and more or less delirium, and which, like many other acute diseases, has a strong tendency to terminate critically.

The *remittent* form, or *mucous fever,* (a name already given to it by several authors), resembles in many of its features the remittent fever of children. It presents one or more daily paroxysms, which occur at irregular periods; is attended at its very outset with considerable derangement of the stomach and bowels, with great prostration of strength, and marked depression of spirits, but not delirium. There is pain of head, often occipital; a sense of weariness over the whole muscular system, particularly that part of it which is most exercised, namely, the extensor muscles; the sleep is disturbed by frightful or annoying dreams; the appetite is impaired; the tongue, sometimes uniformly red, but more generally quite pale, is covered with a very thin, purely white film, and seems made up, as it were, of very minute granules. The bowels are irregularly costive; the motions lumpy and containing sometimes slime, emit a peculiarly disagreeable animal odour. The urine is sometimes scanty and high-coloured; often it is very abundant and limpid, as in hysterical patients. The epigastrium, tender and swollen, is the seat of pain aggravated after taking even the blandest food. Often it feels as if some hard indigestible substance were contained in the stomach. Occasionally there is a sense of vacuity, as though all the viscera were wanting. This is always accompanied with a sense of sinking, and of great weakness. There is almost invariably troublesome cough, seldom attended with much expectoration, and always strikingly relieved by a blister to the epigastrium. The pulse is either quick and sharp, or quick, soft, and feeble.

As the disease advances, the patient becomes emaciated; the countenance careworn; the sides of the nose, the temples, and the region of the parotid glands, of a deep yellow tinge; and the mucous membrane of the nostrils covered with incrustations, beneath which ulcerations often form. The skin dry, and deprived of its naturally clear colour, presents an eruption of mingled papulæ and pustules, which appear on different parts of the body in succession, and terminate generally in very minute scales. In some patients the cutaneous eruption exists during every stage of the disease.

Mucous fever, though very protracted in its duration, for the most part terminates favorably. When, however, a fatal termination is about to take place, the fever invariably becomes *continued,* and gives rise to increased distress of the organs of digestion—to vomiting, diarrhœa, meteorism, &c.; also to heat of skin, suffused watery eyes, low delirium, stupor, diminished organic sensibility, (as evidenced by distention of the bladder, slow respiration, &c.) and, finally, to complete loss of power and tone, occasioning passive (I prefer this term to involuntary) evacuations. In every case thus terminating, I have found the intestinal follicles either in a pustular state surrounded with bright red bases, or more or less advanced in the process of ulceration.

Sometimes mucous fever proves fatal by giving rise to inflammation (apparently

* The mere nausea attendant upon the development of simple fever, can scarcely be regarded as an objection of this statement. It is, in general, a very transient symptom, dependent upon a deranged state of the nervous system.

subacute, yet very rapid in its progress) of one or more of the serous membranes; while occasionally the immediate cause of death is a peculiar species of angina, over which no remedies possess any effectual control, and which carries off the patient generally on the third day. The fauces during life are seen to be swollen, vividly inflamed, and covered partially with muco-albuminous exudation. After death, the inflammation is found to have extended along the lining membrane of the proper air-passages, into the most minute of the visible bronchial ramifications.

With regard to all the circumstances mentioned in these last two paragraphs, a striking resemblance exists between mucous fever, and the remittent fever of children.

The foregoing description of mucous fever is derived from an attentive observation of it during the space of five years, among a class of people who are particularly liable to be affected with it—I mean persons employed at the stocking or lace-frame, who work generally from twelve to fifteen hours daily, and are at the same time both ill-fed and ill-clothed. In such patients, I have known it often to exist for several months together, all the remedial measures that could be thought of serving merely to alleviate the symptoms. In localities where any source of malaria existed, the disease prevailed more generally, was very apt to return after having been temporarily cured, and not unfrequently, *as the patient's strength increased under the use of tonics*, put on a distinctly intermittent type. But it existed in many places in which no probable source of malaria could be detected, and in such cases, its production could be attributed to nothing else than the combined influence of poor diet, and undue confinement in the exercise of a sedentary employment—causes very well calculated to weaken the organic powers. From the enumeration of symptoms, it must be evident how much the digestive organs are implicated; and it is a well-known fact, that almost all abdominal diseases (including even those of the peritoneum) have a strong tendency to remissions and exacerbations.

In many of the patients, worms formed a very troublesome complication, the *ascaris lumbricoides* existing in some, and the *a. vermicularis* in others. It very rarely happened that both these species presented themselves in the same patient. One of the remedies found most useful in the cure of the constitutional disease, was the *liquor arsenicalis;* and this often( but not always) acted as a poison on the worms, which were, in consequence, discharged dead. I could never discover why the medicine, while it exerted such an influence in some cases, failed to do so others.

*Fistula in ano* also was not an unfrequent complication, and I remarked that, of the persons thus affected, a much smaller proportion had tubercles in the lungs, than we usually see to be the case with fistulous patients not labouring under this form of fever. It would hence appear that the fever itself gave rise to the fistula, much in the same way that the tubercular diathesis ordinarily does.

To return to my hospital cases. I admitted four cases of gastro-enteric fever, all of them of the continued type. Of the subjects of them three were in-patients. In the fourth case, the symptoms were unusually mild, and the patient, being the mother of a family, and unwilling to come into the hospital, was seen a few times at her own house.

I shall confine myself to a detail of two of these cases.

M. C. a married woman, æt. 53, admitted Dec. 26th.

*Symptoms.*—Dull aching pain of head; countenance anxious; eyes suffused; great prostration of strength; pain in epigastrium and in arch of colon; urgent nausea and vomiting; diarrhœa, stools being pale, and of a curdy appearance; tongue morbidly red, furred in stripes. Pulse 116, feeble.

Illness had commenced three weeks before admission, and was attributed to anxiety of mind. On enquiring into her circumstances, I found that her diet had been not only poor, but scanty.

Blisters were applied to the epigastrium and abdomen, mild alterative aperients were occasionally administered, and, as soon as the stomach and bowels were brought into a less irritable state, the patient was put upon tonics with a nutritious unstimulating diet. Under this treatment she recovered rapidly, and was discharged cured, on the 24th of January.

CASE 2. E. T. blacksmith, æt. 20, admitted February 21st.

*Symptoms.*—Manner sullen, with a marked air of timidity; countenance very anxious; features sharp; eyes slightly suffused; skin hot; intellect confused; epigastric and right hypochondriac regions tense and tender; bowels slow; tongue very red, furred at root and in centre; pulse 125, feeble; sleep said to be natural.

Eighth dorsal vertebra is very prominent and tender, and spine above has a marked lateral curvature to right side.

Has been out of health for ten months past, but present illness began five weeks ago. A fortnight since he was admitted as an out-patient, labouring under symptoms very similar to those just described, only much less severe.

An ounce of castor-oil was ordered to be taken the morning after admission; and from this period till the 9th of March, the treatment consisted of leeches to the back; small and frequently repeated doses of castor-oil; tepid baths; blisters and leeches to the epigastrium, &c.

March 9th. The breathing to-day being a little hurried, and the chest in consequence examined, right side of thorax is found dull on percussion except above the mamma anteriorly, where respiratory murmur is accompanied with a sonorous râle. Posteriorly (where percussion is dullest) respiratory murmur is not audible, except during a forced inspiration, and then it is heard accompanied with a moist crepitous râle. This side of the chest measures very nearly an inch more than left,* and intercostal spaces are almost on a level with ribs. There is neither cough nor expectoration.

Emplastrum lyttæ amplum lateri dextro adhibeatur.

℞. Hydrarg. submur. gr. iij.
Pulv. opii. gr. ss.
Ext. hyoscy. q. s. fiat pilula nocte maneque sumenda.

Bowels to be kept open regularly by small doses of castor-oil.

13th. Respiratory murmur over right side of thorax more audible; crepitous râle more distinct. Since yesterday has expectorated small ragged masses of dense purulent matter; but save while getting rid of these, *he does not cough, nor has he at any time pain in chest.* Urine high-coloured, with a copious branny deposit, and at bottom a deep pink tinge; bowels freely opened by oil; tongue a little brown in centre; pulse 105, very feeble.

C. C. c̄ ferro lateri dextro adhibeantur, et detrahatur sanguis ad ℥viij.

March 14th. Sumat pilulas ter de die.

Hirudines xij. lateri affecto.

15th. A good night after leeches. Respiration free; cough not urgent; expectoration rather more abundant, similar in character, but with a few minute specks of blood enveloped in the small purulent masses. Bowels relaxed, and the seat of griping pains; urine less high coloured; tongue furred; pulse 100, very feeble.

Ext. opii. gr. ij. h. s. Persistat in usu calomelanos et opii.

16th. Emp. lyttæ amplum inter scapulas.

Extract. opii. gr. ij. h. s.

17th. Slept well notwithstanding blister. Lay the greater part of night on

* The right side of the thorax *in its natural state* very generally measures from an eighth to a quarter of an inch more than the left. This I have ascertained by nearly two hundred comparative admeasurements.

left side, which previously he has not been able to do. Skin perspires freely; tongue moist; pulse 110, soft; urine more abundant, but no deposit; bowels slow.

Ext. opii. gr. ij. h. s.
Ol. ricini ℥ss. primo mane, et repetatur meridie si opus.

18th. Slept 8 hours; tongue moist, furred in centre, red at edges; papillæ elevated; mouth slightly affected with mercurial; one copious evacuation of a bright yellow colour, from oil; pulse 108; blisters discharge freely. Respiratory murmur less audible over affected side of thorax; anteriorly it is heard only immediately under clavicle.

Omittantur *pro tempore* calomel et opium.

Extract. opii, gr. ij. h. s.

Emp. lyttæ infra claviculam dextram.

Ol. ricini cras mane.

March 19th. Ext. opii. gr. ij. h. s.

Ol. ricini cras mane.

20th. Emp. lyttæ inter scapulas.
℞. Pulv. opii. gr. ij.
Hyd. submur. gr. iij.
Confect. q. s. f. pilul. singulis noctibus h. s. sumenda.

22nd. Passed a good night. Countenance much improved; tongue clean; pulse 94; bowels open. Chest sounds clearer on percussion; respiratory murmur more audible, and no longer accompanied with crepitous râle.

24th. Percussion clear over a larger space superiorly; pulse 96, sharp; mouth now decidedly affected; tongue furred, papillæ elevated; one figured stool of a light colour, streaked with green; urine high-coloured.

25th. Respiration hurried; right cheek marked with a circumscribed flush; bowels open during the night; tongue furred; pulse 110; no sediment from urine.

Ext. opii. gr. ij. h. s.

26th. Ext. opii. gr. ij. h. s.

Ol. ricini ℥ss. primo mane, iterumque meridie si opus.

March 27. Breathing natural; countenance free from all expression of suffering. Tongue less red; one stool from oil which did not require to be repeated; evacuation rather light-coloured with very minute rounded masses of fatty matter floating on surface; Pulse, 100; blistered surface of a fiery red colour, and very sensitive.

Extract, opii. gr ij. h. s.
Cataplasma superficiei vesicatæ.

April 2. Setaceum in latere dextro, juxta spinam, inseratur.

7. Seton discharges freely. Percussion clear, and respiratory murmur audible over greater part of affected side of chest, *which now measures a little less than the sound side.* Sleeps well, and begins to have an appetite for food. Pulse, 105. Tongue clean. Bowels slow.

Ol. ricini, ℥ss. primo mane.

℞. Decocti senegæ, ℥j. ter de die.

Ordered *common* diet, having hitherto been on *low*.

From this date he improved uninterruptedly; and on the 9th of May, all traces of pectoral and abdominal disease having disappeared, he was, on account of the spinal disease, transferred to one of the surgeons by whom he was discharged a few days afterwards.

*Remarks.*—I have entered at considerable length into the details of this case, because I believe it to be one of very great interest. The patient on admission, and for some time previously, laboured under a severe attack of gastro-enteritis, for which leeches, blisters, tepid baths, mild aperients, &c., were beneficially employed; but shortly after the symptoms had begun to decline, and while the patient, strictly limited to low diet, was desirous of more substantial food, the breathing became slightly hurried; and on examining the chest, the right side was found to be the seat of extensive disease. The physical signs clearly indicated not merely pneumonia but pleurisy also; although neither then nor afterwards was œgophony heard. But the increased fullness and dullness of that side of the chest (which subsequently became slightly contracted) could be the result of nothing else than pleuritic effusion; while the moist crepitous râle rendered the existence of pneumonia indubitable. The case, indeed, was evidently one of that form of disease, which Laennec terms "pneumonia complicated with slight pleurisy," the latter being merely a consequence of the former.

The thoracic disease must necessarily have existed for some days before the examination was made; and yet even then a slight acceleration of the breathing, scarcely visible to a nonprofessional observer, was all the indication that it furnished of its existence. The patient, though closely questioned, denied having any pain in the chest; there was neither cough nor expectoration before the 13th; and it was two days later still before blood appeared in the sputum. When it did appear, it consisted only of a few minute specks, nor did it shew itself a second time. Who then, *having regard only to the general symptoms*, would have suspected, or at least have satisfied himself, that such extensive disease existed in the chest? The small ragged masses of purulent expectoration were, it is true, very characteristic of pneumonia; but they were so scanty that they would most probably have been overlooked but for the previous discovery of the thoracic disease. Let us suppose, however, that, without the aids of percussion and auscultation, the nature and extent of the affection could have been inferred from a careful consideration of the general symptoms, would the conviction produced by such evidence have been sufficiently strong to lead to that continued decision of treatment, that direct and systematic application of disagreeable remedies—observable in the record of the case, and without which the patient's life must necessarily have fallen a sacrifice to the uncontrolled disease? In what I here say, I am anxious not to be misunderstood. Far be it from me to assume any merit to myself for prescribing a line of treatment, which no one, thoroughly acquainted with the nature and extent of the disease, would have hesitated to adopt. My object in alluding to the point is to illustrate the great importance of attending to the *physical signs of thoracic disease*. This few, if any, are now-a-days bold enough to deny; and yet, *in their practice*, the great majority of medical men still act as if they had no faith either in auscultation or percussion.

Andral, in his admirable "Clinique," has related some cases, very similar to the present, of pneumonia occurring during the progress of gastro-enteric fever, without presenting either cough, thoracic pain, or any other very remarkable symptoms.

In most diseases of the chest, but particularly in pleurisy, blisters would, I believe, be found much more efficacious if they were applied of a *larger size, and more frequently* than they usually are. In the present case, blisters were applied over the whole of the right side of thorax posteriorly and laterally, as well as over part of its anterior surface also. It is a familiar maxim, not to blister in pleurisy, until after the more acute symptoms have subsided; but I have long since satisfied myself that *immediately after venesection*, a blister may be applied, not only with safety, but with the greatest possible benefit, *provided it be sufficiently large*. My plan in severe cases is to cover the affected side from just below the axilla to the inferior margin of the false ribs, and from the spine to the sternum, with one large oblong blister. As soon as vesication commences, the patient invariably feels relieved, and very generally changes his position, turning from the back to the sound side, on which previously he had been unable to lie.*

* Vesication is produced much more certainly and quickly, as well as with less pain, by rubbing the skin, previously to the application of a blister,

Notwithstanding the patient's weakness, I deemed it necessary to have him cupped on the 13th of March, and leeched on the 14th, both of which measures afforded relief.

The calomel and opium, administered for the first time on the 9th of March, and ordered to be taken with increased frequency on the 14th, were continued without intermission until the 18th, when (the mouth being slightly touched) I deemed it advisable, as the patient's strength was much reduced, to omit them for a time. They were resumed on the 20th, one dose being ordered daily, and were finally laid aside on the 24th, at which time, though the mouth was decidedly affected, the stools were light-coloured. This was owing to the additional nightly dose of opium, from which however the most marked benefit was derived. I believe, indeed, that by it mainly was the patient's strength kept up; for in inflammatory diseases, attended with exhaustion of the vital powers, opium is, (as Sydenham long since declared it to be,) the best, and often the only safe cordial.

The state of the bowels required constant attention, on account of some slight traces of the original disease, but in a great measure because of the frequent use of opium. They were regulated almost solely by small doses of castor-oil, frequently repeated. On the 24th of March (the day on which the calomel was omitted) five grains of the alterative powder of the hospital (consisting of hyd. c̄ cretâ, pulv. rhei, and pulv. ipecac.) were given with a view of assisting in the restoration of the biliary secretion. To maintain it afterwards the castor-oil alone was found perfectly sufficient.

On the 2nd of April a seton was inserted, partly with a view to benefit the spine, and partly on account of the pulmonary disease which was not yet quite subdued. It continued to discharge freely during the whole of the patient's subsequent residence in the hospital, nor was it removed on his dismissal.

### Remittent Fever of Children.

Of this disease I treated seven cases among the in, and nine among the out-patients. Of the former class of patients the average age was 10, of the latter, only six years. This difference is to be accounted for by the fact, that patients under 7 years of age are not admitted into the hospital, unless for operation. The average duration of the in-cases was forty-two days; of the out-cases sixty-six days. Of the whole sixteen patients, eleven were cured, three were relieved, one was discharged without benefit, and one died.

I have already alluded to the analogy existing between mucous fever and the remittent fever of children. This resemblance, indeed, is so striking, not merely as regards one symptom, but as regards almost all, that it would be more cor-

---

the strong acetic acid, and mixing up a quantity of the same with the blistering plaster. In this way a blister may be got to act efficiently in from three to five hours, and if, after its removal, a warm poultice be applied for an hour or two, and the part then dressed with *wadding* or carded cotton, scarcely any inconvenience is afterwards experienced. The wadding absorbs the moisture, and affords to the raw surface a soft warm covering, infinitely more comfortable than any greasy application whatsoever. Should the wadding, after some hours, begin to feel hard from the coagulated secretion on its surface, it ought, even though slightly adherent, to be removed and a fresh piece applied.

A great advantage of this mode of blistering is, that persons who cannot, or will not, confine themselves during the time necessary for the common tedious process, may be readily induced to submit to *it*,—seeing that a blister applied at bed-time, will have full time to act without interfering in the least with the engagements of the following day.

rect to consider them as identical, any differences that exist being easily accounted for by the difference of age. Thus in children, in whom the glandular system is so much more active, the disease is always attended with more or less enlargement of almost all the lymphatic glands. In them, worms too, are a more frequent concomitant, although they are by no means uncommon in the mucous fever of adults; as has been shown by the observations recorded in a previous page, and as has been testified by Rœderer and Wagler, by Huxham, and numerous other authors.

But unless under circumstances similar to those in which the class of people formerly specified are placed, adults are not very often affected with mucous fever, while the remittent fever of children is met with in all places, and is of every-day occurrence. From a consideration of these facts we are led to conclude, that confinement, deficient clothing, and poor diet, are to be regarded as its chief exciting causes. The influence of these in producing a weakened and deranged condition of the several organic functions has already been alluded to, and is so easily comprehended that it needs not to be further insisted on.

Dr. Butter's division of the disease into three varieties seems to me a very unnecessary refinement, his *acute* and *chronic* varieties being nothing more than different stages of the same form of disease, while that named the *low* is merely a modification induced by original or acquired weakness of constitution in the patients affected.

The remittent fever of children is so constantly met with, and is so well known, that it would be only lost labour to give here a *general* description of its symptoms. Nor are these sufficiently varying in different patients to induce me to enter into the details of more than three cases. One of these proved fatal, and is selected on that account, the *post-mortem* appearances being highly deserving of notice. But before proceeding to these special details, it may be well to impart some general notion of the plan of treatment pursued, and which was framed chiefly according to the following indications:

1. To improve the state of the secretions. This was effected by calomel, mercury with chalk, sulphate of potass, rhubarb, castor-oil, and (if the expulsion of worms appeared an object of importance) scammony or jalap added to the calomel. But in general active purgatives were not resorted to. The compound calomel pill (in children old enough to swallow pills) was found highly serviceable. To those who cannot take the medicine in this form it might be given as a powder, in which shape I have of late occasionally prescribed it even to adults, having in several instances found the pills to be discharged by stool *perfectly unaltered.* In the first case of this kind that I met with, I could not help thinking that the pill had by accident fallen into the pot which contained the evacuation; but it was not long before a second and a third instance, (occurring with different patients), deprived me of all reasonable ground for such a suspicion.

2. To give strength and tone to the general system.—With this view I administered (the excretions being previously improved) quinine, combined sometimes with ipecacuan, sometimes with mercury and chalk, according as different symptoms prevailed; infusion of gentian or cascarilla with hydriodate of potass, the latter in doses varying from one fourth of a grain to one grain; recently prepared carbonate of iron; nitrate of silver, &c. The last substance here mentioned was prescribed with the double view of strengthening the general system, and of exciting a healthy local influence on the digestive mucous membrane. In every case in which it was used it was found highly beneficial. The dose varied from the twelfth part of a grain to a whole grain. To the younger children it was given in solution, to the older ones in pills. In the latter form it has, I think, a better chance of reaching the intestines undecomposed by the hydrochloric acid and salts of the gastric juice.

Case 1.—J. A., æt. 10, a very intelligent little boy, who accurately described his sensations, was admitted May 6th.

*Symptoms.*—Emaciation, countenance pale, eyes dull, mucous membrane at entrance of right nostril covered with thick incrustations; frequent *irregular* chills, followed by partial flushes; dull heavy pain of occiput; respiration short; action of heart irregular; pain in epigastrium and abdomen; both of which are tumid, the latter tender also; appetite variable; tongue furred, papillæ red and elevated; bowels costive; evacuations light-coloured and very offensive; lower extremities weak, and the seat often of aching pains.

Illness began about two months ago, previously to which time, (but not since), he had passed several round worms.

*Treatment.*—Hyd. submur. gr. iij.

Pulv. jalapæ comp. gr. xx. f. pulvis bis in septimanâ sumend:

℞ Sodæ subcarb. ʒj.

Conf. aromat. ʒiss.

Acid sulph. dil. ʒss.

Aq. menth. pip. ℥vj. f. mist. sumat. ℥ss. ter de die.

May 12th. Pulv. rhei.

Hyd. c̄ creta. āā gr. ij. M. ft. pulvis singulis noctibus sumendus.

25th. Rests badly, starting from his sleep, and fancying he sees frightful images. Tongue clean, but papillæ elevated and red. Has not passed any worms, although powders have operated with moderate briskness.

℞ Argenti. nitrat. gr. vj.
Extract. hyoscyam. q. s. misce optime et fiat massa in pilulas xxiv. dividenda, e quibus una sumatur ter in die.

Persistat in usu pulverum purgantium, ut etiam pulverum alterativorum, non misturæ.

Under this treatment the little patient's health soon began to improve; his appetite became steadily good; his heart ceased to beat irregularly; and his sleep was no longer disturbed by dreams. On the 11th June the pills were omitted, and he was ordered, instead, one ounce of decoction of bark thrice daily. On the 17th of the same month he was discharged cured.

Case 2.—A. P., æt. 12, admitted Feb. 7th, 1837.

*Symptoms.*—Countenance pallid, with a livid circle under eyes; flesh wasted; skin dry, presenting on extremities (particularly in the neighbourhood of joints) numerous minute scales; glands of neck enlarged; frequent irregular chills; head-ache, accompanied often with drowsiness; palpitation of heart; nausea; abdomen full; umbilical and hypogastric regions the seat of constant pain; tongue clean, breath fetid; bowels costive; pulse 60, feeble.

Disease has existed twelve months, during the greater part of which time, she has been subject to fits, attended with loss of consciousness and with slight convulsions, but not with foaming at the mouth. They come on at irregular intervals, and are preceded and followed by extreme depression of spirits, leading even to tears. Soon after the commencement of fits, she began to pass round worms, of which 31 were evacuated in a period of six months.

*Treatment.*—Hyd. submur. gr. iv.
Pulv. scammon. gr. v.
Pulv. jalapæ co. gr. xxv. f. pulvis cras mane sumendus, bisque in 7na. repetendus.
Diebus a purgatione liberis sumat ægra ter in die,
Ferri Carb. recentis gr. x. ex theriacâ.

Feb. 11th. A fit on the night of admission. Bowels four times acted on. No worms.

14th. No fit since last report. Countenance looks better.

16th. Took the powder yesterday and passed, during its operation, a large ascaris lumbricoides.

22nd. Fits have not returned. General health improves, but pain in umbilical and hypogastric regions continues. Pulse until yesterday did not rise above 65: it is now 74.

The umbilical and hypogastric pains still continuing, together with a dull heavy sensation in occiput (symptoms which when they occur together, always I believe, indicate the existence of worms in the alimentary canal) I prescribed, on the 6th of March, half an ounce of ol. terebinth. and the same quantity of castor-oil, made into an emulsion with liquor potassæ and peppermint water. It did not bring away any worms, but a similar dose given on the 13th expelled a large lumbricoid worm, after which the abdominal pains ceased, and the patient's health improved rapidly. On the 16th another worm was discharged, without the operation of any purgative, and on the following day quinine was prescribed instead of the carbonate of iron. Eight days afterwards the patient was discharged cured.

*Remarks.*—Although worms are by no means to be regarded as the cause of mucous fever, yet they often give rise to so much direct and local, as well as sympathetic and constitutional irritation, that their expulsion becomes an object of very considerable moment. In such cases one is justified in departing from his usual caution, and prescribing instead of mild aperients, the more active and even drastic purgatives. But he must watch anxiously the effect of every dose, and in case the fever should be aggravated, or the abdomen rendered tender, he must at once intermit the use of purgatives, and have recourse to a soothing plan of treatment; for the continued presence of the worms is, under all conceivable circumstances, a thing much less to be dreaded than inflammation of the mucous follicles, to which active purgation might in certain states of the mucous membrane very readily lead. But if, as in the present case, the symptoms improve after each exhibition of the purgative, there can be no doubt as to the propriety of continuing to repeat it, as long as there is good evidence that the worms are not yet wholly expelled.

The circumstances most deserving of notice in this case are, the very large number of worms passed previously to admission; the peculiar fits occasioned partly by them, but partly also (we cannot doubt) by the accompanying derangement of bowels, for they ceased after the first purge although no more worms were thereby expelled; the depression of spirits, very similar to what takes place in hysteria, and extremely unusual in one so young; and lastly the low state of the pulse, which for several days after admission did not rise above 60 strokes in the minute, although its general range in this disease is considerably above 100. Is the strange fact here recorded attributable to a sympathetic derangement of the cerebral functions, of the existence of which the fits already alluded to afforded undoubted evidence?

The anæmic state of the patient induced me to try first the carbonate of iron. Had the fits not ceased very soon, I should most probably have substituted for it the nitrate of silver.

CASE 3. E. T. æt. 6,* admitted 21st of March, 1837.

*Symptoms.*—Great debility; countenance pale; mucous membrane of nostrils

* This patient though under the age specified by the rules of the hospital, was admitted as an in-patient, both on account of the serious nature of her disease, and because her mother, being at the time a patient in the hospital, could pay her every necessary attention.

covered with incrustations, which the little patient often picked off; skin dry; one chill (almost amounting to a rigor) each afternoon, and followed by slight pyrexia; cervical glands on both sides much enlarged; abdomen tumid; occasionally the seat of pain; right lower extremity painful and at times partially paralysed; tongue reddish; bowels slow; motions dark-coloured.

Has been out of health sixteen months, during which time she has passed three round worms.

*Treatment.*—Pulv. scammon. co. gr. xij. cras mane, et postea oleum ricini si opus.

22nd. A small lumbricoid worm voided.

From this date until the 13th of April, three purges of calomel and scammony in moderate doses, and followed, when necessary, by castor-oil, were prescribed without bringing away any more worms; and the little patient took besides, twice a day, five grains of recently-prepared carbonate of iron in treacle. For a time her appetite seemed to improve, and her countenance looked better, but on the 13th of April, she complained of pain in the epigastrium, seemed averse to food, and vomited the tea, &c. taken at breakfast. A blister was applied to the epigastrium.

April 14th. Respiration hurried and laboured, and performed with a loud, thick, muffled sound; eyes watery, slightly suffused, countenance pale, lips parched, complains of pain in throat which on examination is found to be highly inflamed, the inflammation extending from about commencement of posterior third of palate backwards over velum, uvula, amygdalæ, fauces, and as far down as the eye can reach. It is of a bright red color and the mucous membrane appears as if it were raised up by subjacent effusion. The fauces present some pretty large patches of white, and apparently very tenacious muco-albuminous effusion. Skin hot; tongue dry; bowels open yesterday; pulse 130.

℞. Hyd. submur. gr. j.

Pulv. ipecac. gr. ij. f. pulvis. 4ta quaque hora sumend.

℞. Argenti nitrat. ℈ss. Aquæ distillat. ʒiij. ft. solutio palato ac faucibus libere applicanda.

Hirudines tres gutturi.

15th. The leeches were applied to the throat soon after visit yesterday, but the patient becoming delirious, and apparently unable to swallow, only one powder was given. An attempt was made to apply the solution, but with little success. The patient, indeed was rapidly sinking, and it was evident nothing could arrest the progress of the disease. During the early part of the night, she threw her arms about, tossed the head from side to side, and uttered low plaintive moanings. Towards morning, the respiration became slow, and the patient comatose, in which state she remained four or five hours, and then died without a struggle.

*Inspection.* Veins on surface of cerebral hemispheres distended, containing soft dark coagula floating in clear serum.

Fauces and pharynx partially covered with a thin layer of viscid muco-albuminous effusion—mucous membrane beneath *slightly* inflamed. Œsophagus healthy.

Lining membrane of larynx of a light rose-colour; that of trachea deeply stained and covered with a thin sanguinolent mucus—the line of distinction between them abrupt and well-marked. Bronchial tubes half filled with red frothy fluid; their lining membrane inflamed, but not so deeply as that of trachea.

Inferior lobe of right lung much congested; upper lobe so strongly adherent to costal pleura, that, in separating them, a rounded mass of pulmonary substance, more than half an inch in diameter, was torn away, and remained attached to ribs. Two ounces of serum in left pleural sac. Bronchial glands converted into a soft white substance very like cream-cheese; but conversion in some of them incomplete, shreds of the glandular substance still remaining, and being surrounded with the cheesy matter hardened so as to form sacs, several of which were more than half empty. Two of the glands were as large each as a pigeon's egg.

A coagulum consisting partly of colourless fibrine, and partly of dark blood in right auricle, and extending into right ventricle. Uncoagulated, thin, black blood in left auricle.

Anterior surface of liver marbled with irregular yellow streaks, extending chiefly backwards, and found on incision to penetrate the whole substance of liver. Gall-bladder distended with dark green bile, which had begun to exude upon the liver and adjoining viscera. Mesenteric glands enlarged.

Lining membrane of duodenum and upper part of jejunum smeared over with yellow bile. The former presented two patches thickly studded with minute dark green points, the matter causing which appearance existed seemingly in the submucous cellular tissue. The lower part of the duodenum contained four small round worms, rolled up into a mass, the mucous membrane near them being in a state of high vascularity. The *glandulæ solitariæ* of the duodenum were much enlarged, of a pearly white appearance, and without any surrounding vascularity. Those of the jejunum and ileum had a pustular appearance, with elevated inflamed bases, and were for the most part encircled with radiating vessels. One, with which an ascaris lumbricoides was in contact, was as large as a split-pea, and was deeply ulcerated in its centre. On the side of the intestines opposite to the attachment of the mesentery, the mucous membrane throughout the whole of the jejunum and ileum presented numerous elevated longitudinal types of a pale fleshy appearance, measuring from half an inch to an inch in length.

The spleen was larger than natural and firm.

The other organs were healthy.

*Remarks.*—The morbid appearances above described, coupled with the history of the case, afford abundant matter for reflection.

Did the purgatives that were given aggravate the intestinal disorder? It is difficult to conceive how they could fail to do so, yet it is certain that the patient's health slightly improved for a time under their use. But is it not probable that for some time after admission, the intestinal glands were merely enlarged (as those of the duodenum still remained), and that they put on the appearances discovered on dissection only a few days before death? This is the view of the matter which I am inclined to take, when I consider the benefit apparently derived from the purgative and tonic plan of treatment, as well as the very short period prior to death at which the fever began to present inflammatory symptoms, and to assume a continued type. Nor can we wonder that pustules should form so rapidly on the mucous membrane, if we only reflect how quickly they sometimes develop themselves in the skin. But, conceding the correctness of the above notions, may it not still be argued, that the purgatives and tonics (particularly the former) *led to the production* of so much inflammation? That they were calculated to increase in the mucous membrane any disposition to inflammation that might exist, cannot be doubted; but that they were the sole or even principal cause of it is, in my opinion, incredible. The intestinal inflammation and eruption of pustules are, I believe, as much essential parts of the acute form of gastro-enteric fever, as those of the skin are essential parts of any of its eruptive pustular diseases. Yet, notwithstanding all this, it is unquestionable that whenever there is reason to believe that such a tendency exists in the digestive mucous membrane, all active purgatives ought to be carefully avoided. The present case, therefore, which is so well calculated to enforce this important practical caution, is purposely placed by the side of the preceding one, in which a succession of active purgatives was exhibited with very decided and unmixed benefit.

Are there any sure grounds of distinction by which we can discriminate accurately between these two classes of cases? And if not, are we to forbear altogether from purgatives, and thus give up the good which so often results from their use, rather than incur the risk of their occasional pernicious consequences?

To the former of these questions I would answer that *in general* an accurate distinction may be made by a careful consideration of *all* the symptoms. If the fever lay aside the remittent, and assume the continued, type, if the tongue become morbidly red, the appetite much impaired, the skin hot, the abdomen tender, the head painful, the eyes glassy or suffused, and the temper unusually irritable, there can be no doubt that inflammation of the intestinal mucous follicles already exists. And even though only two or three of these symptoms present themselves, and that also in a slight degree; yet if they are aggravated after the administration of even a mild purgative, there can be no question as to the propriety of guarding carefully against the use of every thing stimulating. But redness of the tongue alone by no means contra-indicates the use of purgatives. In the last case but one here recorded, in which very active purgation was employed with such decided advantage, the tongue was much redder than it was in the fatal case.

It being thus shown that in general the two classes of cases (viz. that class in which purgatives are useful, and that in which they prove injurious by aggravating the intestinal inflammation) may be accurately distinguished, it follows that to proscribe purgatives altogether from the treatment of the disease, would be to deprive ourselves, without a sufficiently good reason, of one of our most effective means of cure.

Notwithstanding the highly enfeebled state of the patient's constitution, the inflammation of the throat very speedily after its commencement attained to an exceedingly high degree of intensity, producing an effusion of plastic lymph. John Hunter was of opinion that this form of mucous inflammation "has something of the erysipelatous disposition,"* an opinion which he formed, no doubt, from a consideration of the rapidity with which it extends along the mucous surface. In the present case it spread very rapidly, beginning in the throat, and extending in a few hours down the trachea, and ultimately into the most minute of the visible bronchial ramifications. Yet the breathing, though altered in the manner described, had nothing of the croupy sound, and there was little, if any, of that agonizing distress which patients labouring under genuine croup experience. The absence of such symptoms is explained by the fact that the lymphy exudation did not extend lower than the lowest part of the fauces. The proper air-passages, though highly inflamed, did not throw out coagulable lymph.

Sydenham, in describing angina, says, "æger spiritum per nares ducere non valet,"† and in fact the respiration in the present case was such as one might pretty accurately imitate by breathing rather forcibly, and compressing at the

* This great man, whose writings, long considered in part unintelligible, have become better understood and more appreciated in proportion as we have advanced nearer to him in pathological knowledge; the republication of whose works at the present day affords a cheering omen of what may be expected from the philosophical spirit which now pervades every rank of our profession—believed that the erysipelatous inflammation produces results the very opposite of those to which common inflammation gives rise; namely, in the serous membranes suppuration, and in the mucous the effusion of coagulating lymph.—*Vide "Treatise on the Blood," vol.* 1, *p.* 427. *London,* 1812.

† Opera Universa Medica, p. 248. Lipsiæ, 1827.

same time both nostrils. Huxham, in his admirable delineation of *angina maligna*, has the following passage: "respiratio reddebatur difficilior, *cui stertor et sibilus accedebant*, qui ægro tum jugulare videbantur. Vox rauca et obtusa illi erat simillima quæ a veneris in faucibus ulceribus oritur: loquelæ respirationis—qui sonitus iste ita erat peculiaris, ut homines, qui cum ægrotis versati erant, facile morbum agnoscere possent sonitu illo miro. Sonum illum acutum, latrantem, quem sæpe in angina inflammatoria." (Croup) "percipimus *nullus meorum ægrotorum reddidit*."*

It is worthy of remark that the larynx was very little affected, its natural colour being scarce perceptibly changed, while the parts both above and below presented evidence of high inflammation. This is a very common, though, as far as I am aware, unexplained phenomenon in such affections of the air-passages. And yet it is possible that during life the larynx may have been more inflamed than its appearance after death would lead us to imagine; for on the day preceding that on which the patient died, the throat (unless in those parts covered by coagulable lymph) had exactly the appearance which the skin presents when it is affected with very acute phlegmonous inflammation; but when the parts came to be inspected after death, the bright red colour had faded, and only a very slight blush of inflammation remained.

That the inflammation of the air-passages was the immediate cause of death, admits not of doubt; but whether it should be regarded as an accidental affection totally unconnected with the intestinal disease, or as originating in the same state of the constitution which produced this latter, and therefore closely alied to it by nature—is a question not so readily solved. I am inclined however, to view it in the latter light, chiefly because all the *precisely similar cases* I have seen, have occurred in such patients. In two instances however the subjects of the affection were adults, and the intestinal lesions were merely inferred from the symptoms; for in neither case was a post-mortem examination obtained. In both, death took place on the third day from the first feeling of uneasiness in the throat, the fever after the first few hours having assumed a well-marked typhoid type, and (in one of the patients) petechiæ intermixed with very minute pustules having appeared on the lower extremities. Both these patients were of delicate constitutions, and their strength had been exhausted by previous disease.

The occurrence of membranous angina in scarlatina, measles, gastric fever, erysipelas, &c. has been noticed by several writers, and proves most satisfactorily that the local inflammation is not, generally speaking, an independent affection; but only part and parcel of the constitutional disease. The present observations will, I hope, attract some attention to its frequent existence as a complication of remittent fever, both in children and adults. Huxham evidently described such a complication in his account of the epidemics of April, 1734;† but with the characteristic symptoms of it, he mingles those of simple amygdalitis, in such a way as to prove that he confounded strangely these two widely distinct affections.

---

* Huxhami Opera, p. 592. Lipsiæ, 1829.

† Op. Citat. p. 93; also p. 175, in which he speaks of *a species of angina supervening on chronic diseases and slow fevers*, and which almost invariably terminates fatally. But in his description of the symptoms he says: "nec tumor est extra, nec in faucibus aliquid conspicuum, aridæ sunt quodam modo et pallidæ." Do mere dryness and paleness of the fauces then constitute a species of angina? The truth is, Huxham, as well as all the older authors, so confounded the several species of angina, that they often assigned to one, the symptoms belonging to another and very distinct species. In the work just quoted, p. 173, there is very accurately detailed, under the head of angina, a case of undoubted

In the Medico-Chirurgical Review for April, 1827, there is extracted from Dr. Hamilton's work on the management of children, an account of a "modification of sore-throat in children," which seems to have been no other than that here treated of; although, in some minor points, Dr. H.'s description of the disease is different from that which my own observation would lead me to give of it. I have no doubt, however, as to the identity of the affection, which appears to have proved not less frequently and rapidly fatal in Dr. H.'s experience than (I regret to say) it has in mine.

Is the form of angina, described in the foregoing paragraph as complicating certain diseases, infectious? I do not believe that *it* ever is, although the disease of which it is a complication (as for instance scarlatina) may be, and often unquestionably is. But when it presents itself during the progress of low remittent fever, whether in children or adults, neither it nor the general disease is, in my opinion, infectious. In this form it has often been known to prevail for a season (generally either in the Spring or Autumn) in some particular neighbourhood, giving rise to the idea of contagion; and passing sometimes under the name of angina maligna, an affection *the individual and separate* existence of which I am very much disposed to question.* Sometimes, again, it has been confounded with *scarlatina maligna*, the minute pustular, and, occasionally, petechial eruption on the skin, together with the frequently simultaneous prevalence of actual scarlatina, imparting to such a classification of it a very strong semblance of truth. But in such instances, instead of regarding either the local inflammation or the fever as contagious, we ought to look upon them as the conjoint result of endemic predisposing, and epidemic exciting causes.

The great length at which I have dwelt on membranous angina, as a complication (and almost invariably a fatal one) of remittent fever, is not, I believe, unsuited to the importance and comparative novelty of the subject. I shall now resume my remarks on the morbid appearances discovered on dissection.

The congested state of the vessels of the brain, together with the existence of black blood in the heart, particularly in its right cavities, (in which it had coagulated), demonstrated, what the symptoms during the last hours of life had indicated, impeded circulation through the lungs, as well as imperfect decarbonization of such portion of the blood as did pass into the greater circulation. The blood found in the left auricle, must have permeated the lungs the moment before life ceased; but its vitality seems to have been lessened rather than increased thereby; for while that in the right auricle had coagulated with some degree of firmness, *it* remained perfectly liquid, and was as black as it is ever seen to be in the most putrid diseases.

The state of the bronchial glands deserves attention. The cheesy matter must

---

laryngitis, which proved fatal in 36 hours, the operation of bronchotomy, at which Huxham had hinted, but which had been strongly discountenanced by the by-standers—"id vero quasi esset hominem jugulare, non servare, mussitabant adstantes"—not having been performed. This case immediately occurred to my mind when I read Dr. Cheyne's statement (Cyclop. of Med. art. Laryngitis) that the case of General Washington was, as far as he knew, the first accurately recorded one in the annals of medicine.

* In stating this opinion, I am glad to avail myself of the authority of Sydenham, who again and again declares that the affection of the throat, is only a symptom, while the fever is the essential and individual disease. Op. Citat. pp. 223 and 249. It is but just however to add, that this illustrious author took the same view of scarlatina, pleurisy, pneumonia, rheumatism, &c. in all of which the local inflammation, according to him, is nothing more than a complication or result of the constitutional disease. The same doctrine is taught also by the celebrated John Brown, in his *Elementa Medicinæ*.

have began to form on the circumference of the gland, and must have continued to be deposited by the vessels of the gland itself, until, by its pressure, it cut off their communication with the surrounding parts. After this no further vital changes could take place in the body of the gland, so that any shreds of its structure that remained unabsorbed, must afterwards so continue.

In reverting to the treatment of the present case, there is found but little worthy of comment, with the exception of the exhibition of purgatives, on which I have already spoken freely. When the inflammation of the throat commenced, knowing the rapidity with which the affection usually runs its course, I felt that it was incumbent on me to use every exertion to check it. I accordingly prescribed leeches to the throat, the internal application of a solution of nitrate of silver, and calomel with ipecacuanha, the latter in two-grain doses, intending that it should at first excite vomiting. Had the patient been more vigorous, and had I been less apprehensive about the condition of the digestive mucous membrane, I should have prescribed tartar-emetic instead of the ipecacuanha; for notwithstanding M. Guersent's preference of the latter in simple croup, I believe that it is by no means equally efficient with the former in arresting the progress of inflammation. In the present instance, there was no opportunity of trying its powers, for the patient sank so rapidly that only one dose was given.

The same may be said of the caustic solution, which it was found impossible to apply effectually. But in my own experience all the local applications employed have invariably disappointed my wishes. In some cases, indeed, they have appeared to aggravate, and cause an extension of the inflammation. There is one, however, of the effects of which I can say nothing from experience—I mean undiluted muriatic acid, as recommended by M. Bretonneau. I have always shrunk from its use, and the more so as the recommender of it subsequently adopted, by preference, another local application, to wit, powdered alum reduced to a paste with water.

The application of a mineral acid to the throat when inflamed, was nothing new when proposed by M. Bretonneau, the sulphuric having long before been recommended by Sydenham. He prescribed it, however, mixed with honey of roses, "ad summum acorem." This appears to me a less objectionable method of employing so powerful an agent.

To conclude.—From all that I have witnessed of this formidable affection, I should be inclined, in any cases of it that may afterwards occur to me, to place more reliance upon tolerably large doses of quinine, repeated at intervals of two or three hours, than on any other remedy or set of remedies whatsoever. There still however remains a wide field for observation as regards both the treatment and the nature of the affection; and such, in my opinion, is the importance of the subject, that I should not consider I had acquitted myself properly, either as regards the profession or the public generally, if I had treated of it less comprehensively than I have done.

### BILIOUS REMITTENT FEVER.

The disease so named is, in my opinion, nothing more or less than a quotidian intermittent of the severest type. The great length of the paroxysm, and the unusual violence of the symptoms, leave the system neither time nor power for the formation of a perfect intermission.

The causes which operate this marked difference are two-fold; first, an over-full and inflammatory habit of body, accompanied for the most part with excessive derangement of the chylopoietic viscera; and, secondly, the superaddition of cold as an exciting cause, the system being already under the latent influence of malaria. Sometimes it happens that those agents affect the body simultaneously, on a first exposure to miasmatic influence. Very often the action of cold is augmented by that of moisture also, both combining to render the skin imperspirable, and thus increasing the inflammatory tendency.

To the two causes above specified, we may with good show of reason, add a third; but as *they* are matters of observation while *it* is necessarily hypothetical, I have thought it better not to conjoin them. It is, an unusually strong dose (if I may use such an expression) of the malarious poison.

The true nature of this fever is clearly demonstrated by the following facts:

First—It not unfrequently begins as a distinct tertian-intermittent, then assumes a quotidian, and, soon after, a remittent type.

Secondly—By blood-letting, mercurial and saline purgatives, and a cooling regimen, it may often be reconverted into its primary intermittent form.

Thirdly—It is met with almost exclusivdly in those localities and seasons, in which intermittents prevail.

Fourthly—It cannot be perfectly cured without the use of those medicines, particularly bark, which are necessary in the treatment of intermittents generally. Indeed it often requires, as Sydenham long ago remarked, a larger quantity of bark for its cure than the common intermittents do.

The older writers do not often employ the term remittent fever, but they treat accurately of the disease under other names. Thus some speak of it as merely a variety of the tertian or quotidian intermittent. Others, again, consider it under the distinct title of semitertian or the Greek synonyme, Hemitritœus, although the general definition of this form of fever is seldom strictly applicable to it.* The detailed descriptions however, are such as cannot be mistaken (vide Spigelius, Hoffmann, Baglivi, &c.) Almost all mention its true relationship to the family of the intermittents; its seeming difference in the assumption of an imperfectly continued type; its frequency in plethoric, over-fed patients; the marked severity of its symptoms; the universally disordered state of the chylopoietic functions; the firmer and more tenacious state of the blood than in the simple intermittents; the frequent existence of phrenitis, pleurisy and pneumonia, as complications; in fine, its dangerous nature and frequently fatal termination, (vide Celsus, Galen, Baillou, Spigelius, Sydenham, Hoffmann, Baglivi, Boerhaave, Huxham, &c.)

Bilious remittent fever prevails chiefly in the Autumn, and is met with almost exclusively in low marshy, or thickly-wooded parts of the country. The great agricultural improvements which have taken place in this country during the last half-century have diminished very much the frequency of its occurrence.

With one exception, all the more severe cases that I have met with in this neighbourhood, have occurred in robust men, employed in cutting down the thick

* The Hemitritœus of Celsus, which is the same as the semitertian of Hoffman, Cleghorn, &c., is characterized by a daily return of the fever, with a remission considerable on alternate days, but scarcely marked on those intervening. Hence it has been regarded by some authors as a compound of the quotidian and tertian types. But this latter, strictly speaking, is *the simple original type*; the quotidian itself, as well as all the others, being merely modifications of it. A robust plethoric constitution, local inflammation, &c., favour the production of the quotidian form; while a shattered constitution, impure blood, chronic congestions and enlargements of the viscera, &c., tend, on the contrary, to make the disease assume the quartan type. Hence the long duration of the cold stage in this last.

I have remarked, in the text, that the general definition of the semitertian is seldom strictly applicable to the remittent fever of this country, the remissions of which do not, in general, differ so much on alternate days. Yet I have met with several cases in which (particularly towards the decline of the fever) the difference was very considerable.

underwood, which abounds so much in East Kent. In such patients, the disease has always suddenly supervened on exposure to rain and cold, and has been attended with violent inflammatory symptoms. Pneumonia and bronchitis have been more frequent complications than pleurisy, and delirium has been present almost invariably. This symptom is, *generally speaking*, independent of actual disease in the brain, and is more marked in those cases in which gastro-intestinal mucous inflammation, pleurisy, or pneumonia, (particularly the two former), exists as a complication. The older physicians thought differently, and, regarding the delirium as the result of a genuine phrenitis, were very much guided in their prognosis by the degree of violence to which it attained. Thus Sydenham, speaking of the fever which prevailed in the Autumn of the year 1678, says, "Postea vero febris hœc eo usque etiam præter indolem suam incrudescebat, ut intermissionis loco *remissionem tantum ægro concederet, et magis in dies ad continuarum speciem accedens, tacto cerebro tandem haud paucos extingueret.*" The truth is, the violence of the delirium, the approach of the fever to the continued form, as also the actual danger, are all proportionate to the extent and severity of the existing inflammation, whether in the chest or abdomen. I do not mean entirely to exclude cerebral and meningeal inflammation, but when one or other of them does exist, it is, I believe, very often of secondary occurrence.

When the disease is fully developed, it generally happens that a single paroxysm with the remission, occupies the whole of the twenty-four hours, and, in very severe cases, even a longer period.* But I have met with instances in which there were two distinct daily paroxysms, one being much less violent and of shorter duration than the other. In these the patients were less robust, and the disease not attended with such highly inflammatory symptoms.

An extremely copious secretion of bile, though by no means an invariable, is yet a very common symptom of this disease. The conjunctivæ are most generally gorged, and the whole of the skin is deeply tinged with it. The quantity discharged by stool is in some cases enormous. I have known from three to five rather copious evacuations, consisting almost altogether of bile, to be passed daily for the space of more than a fortnight. This profuse secretion indicates necessarily an over-active state of the liver, induced, no doubt, by previous congestion in the portal system, as well as by the imperfect elimination of carbon from both the skin and the lungs. It occurs generally towards the decline of the fever, which always abates in consequence, but never, according to my own experience, undergoes a completely critical termination. Under such circumstances I have ventured to give the bark, without any disadvantage. Indeed, but for this potent remedy, the excessive biliary secretion might continue for a very long period, each cold fit renewing the portal congestion, and thus, affording materiel for a fresh supply of bile. But while thus administering the bark, it is always necessary to keep a strict eye to the state of the digestive mucous membrane, particularly to that part of it which lines the small intestines. Hoffman, as also Huxham, recommended nitre in combination with the bark.

The fever sometimes undergoes an imperfect critical termination by an abundant hæmorrhage from the bowels. A corpulent female whom I attended in the

* Cullen asserts that both in intermittent and remittent fevers, the whole paroxysm (including the intermission or remission) never extends beyond the period of twenty-four hours. "There is not," he adds, "one observation in physic in contradiction to this." Celsus, on the other hand, describes the paroxysm of the hemitritœus as extending to between twenty-four and thirty-six hours. "Porrigitque febris inter horas viginti-quatuor et triginta-sex." I have certainly seen the paroxysm of remittent fever extend beyond the nycthemeral period; but in all the cases in which this was witnessed the accession of the paroxysms was irregular, and the disease might very easily have been mistaken for continued fever.

Autumn of 1836, and who had laboured under the disease for several weeks before I saw her, was attacked, a few days after taking the bark, with intestinal hæmorrhage, the quantity of blood passed in twenty-four hours exceeding four pints. She recovered rapidly. This termination of the disease is noticed by Hoffmann in the following terms; " quando fluxus oboritur vel biliosus ac pituitosus, *vel etiam cruentus plane, bonum, et morbi solutionem nunciat.*" (Opera omnia, t. ii. p. 41. Geneva, 1748.)

As regards the treatment of bilious fever three leading indications present themselves :—First, to regulate the state of the stomach and bowels ; secondly, to relieve plethora, whether relative or absolute, and reduce any existing local inflammation; thirdly, to prevent the accession of the paroxysms by the administration of bark or quinine. The first two indications may be reduced to practice simultaneously ; the fulfilment of the third should never be attempted until after the others have been duly attended to.

But, on the other hand, I have known much mischief result from unnecessary delay in prescribing the bark. Indeed I have met with cases in which it had not been given at all, the disease approaching the continued type so nearly that it had been mistaken for simple inflammatory fever. This error had arisen of course from highly culpable inattention on the part of the medical attendants. The patients, as might be expected, had never completely recovered, but had remained for months, and in one case for a whole year, labouring under a low nervous fever, accompanied with extreme depression of spirits, irregular chills, and a highly disordered state of the chylopoietic viscera.

The remedies found most useful in the treatment of bilious remittent fever are, in the formative stage, one or two emeto-cathartics (one grain of tartar-emetic to six or eight drachms of Epsom salts for a dose); when the disease is developed, bloodletting, both general and local—the latter effected by cupping-glasses to the epigastric or right hypochondriac region ; mercurial and saline purgatives ; antimonial diaphoretics (ʒss. ad ʒj. of antimonial wine in ℥j. of distilled water, every two, three, or four hours, according to the intensity of the symptoms) ; and, as soon as the more acute symptoms have subsided, the bark or quinine during the remissions, the antimonial medicines being still continued during the paroxysms. Purgatives also will still at times be required. It is singular that Sydenham, Hoffmann, and many others of the older physicians, should have been so much prejudiced against the use of purgatives in this disease. Even Huxham did not venture upon any but the very mildest. In those cases, indeed, in which positive inflammation of the gastro-intestinal mucous membrane exists, purgatives must prove injurious ; but such inflammation is, in my opinion, *very frequently a consequence of neglecting to remove the primary congestions*, by establishing and maintaining an active state of the different excretory organs.

When any local inflammation is detected, it ought, of course, to receive appropriate attention and treatment, and until it has been removed, the bark must be withheld.

Opium, which is very seldom admissible during the active state of the disease, is often extremely serviceable towards its decline. There is then met with in many patients, a strong mental delusion, attended for the most part with sleeplessness, and which yields to opium much more readily than to any other remedy. This symptom has been noticed by Dr. Jackson, and by Dr. Jos. Brown, the latter of whom states (Cyclopæd. of Med., art. Remittent Fever) that it is not confined to the advanced stage of the disease, but exists from an early period. With this statement my own experience does not correspond ; but I have no doubt of its general correctness when I consider the opportunities for observation possessed by the excellent physician who makes it. I think it right therefore to add, that it is only when the symptom exists *towards the decline of the fever*, that I recommend opium as a remedy for it.

The only case of the disease treated by me in my hospital practice illustrates, in a very striking manner, the good effects of opium when given at the time, and under the circumstances above described.

CASE 1. J. B. æt. 17, of a sanguineous temperament, by occupation a woodman, admitted January 10th.

*Symptoms.*—Intellect confused, so that he is incapable of answering correctly the ordinary questions; countenance of a deep violet colour; eyes slightly suffused and watery; skin hot, of a brownish yellow tinge; respiration laboured; epigastrium full; tongue thickly furred; pulse 130, soft and very feeble; mucous râle over whole of chest.

The person who accompanied him stated, that the disease had commenced four weeks before, in consequence of the patient's being drenched with rain while employed working in the woods; that it was ushered in by a chill which occurred soon after noon, and lasted several hours; that then high fever set in, which, according to her statement, had continued ever since. No medical treatment had been employed.

Balneum tepidum quamprimum, et postea hirudines sex infra claviculas.

Ol. ricini, ℥j. cras mane.

For four days after admission the treatment was confined to regulating the bowels, and improving the secretions and excretions generally, by means of aperient alteratives and tepid baths. During this period the remittent character of the fever was clearly ascertained. A chill took place each afternoon, and was followed by pyrexia which lasted until between ten and eleven o'clock on the following morning. Then partial and slight perspirations ensued, and a remission of from three to four hours' continuance. But the febrile paroxysm, at the end of the four days, had become shorter and milder, while the period of the remission was on the increase. A large quantity of bile had been discharged by stool; the epigastrium had become less full; the skin much more perspirable; the tongue less coated; and the pulse fuller and slower, being 96 in the remission, and from 115 to 120 in the paroxysm.

During the period above-mentioned, the patient had remained in the same confused state of mind which he presented on admission, his countenance being dull almost to vacancy, and his answers to the ordinary questions, though to a certain extent just and pertinent, being yet contradictory, and having reference, in part, to something else than the points on which he was interrogated. But on the morning of the 14th, he was found out of bed, and attempting to dress himself, and on being questioned as to what he was about to do, he stated, that his two children were dead, and that he must go home to his wife who was distracted at losing them. (It should be borne in mind that the patient was only seventeen years of age and unmarried). He was with difficulty persuaded, or rather forced, to return to his bed, where he continued to lament the death of his children, and to speak feelingly of the sorrow of his wife, until an early part of the afternoon, when (the other patients having gone to dinner) he arose, dressed himself, and forming his linen, &c. into a bundle which he took with him, found his way down stairs, and was issuing from the outer door of the hospital when he was stopped by the porter. Being in the house at the time, I was called to him, and found him greatly exhausted by the efforts which he had made, his pulse being so feeble and quick that it could scarcely be counted. Though much agitated, he informed me coherently of his children's death, and his wife's state of unsupported affliction, he being absent from her. Tears filled his eyes as he spoke, and his countenance was strongly expressive of the grief which he so feelingly uttered. He was assisted back to bed, and a person was appointed to watch constantly by him, as long as the delusion under which he laboured continued.

I directed the attention of the pupils who were present to the peculiar nature of this delusion, so different from the common delirium of fever, in that the imagined occurrence was described circumstantially, and with all the seemingness of reality, the passion of grief also, *which it was naturally calculated to excite*, being actually and indeed strongly felt. I explained to them that such a delusive impression, leading at once to consistent mental processes, and appropriate moral emotions, could be regarded in no other light than as a true *monomania;* and after stating the frequency of its occurrence in bilious remittent fever, I expressed my hope of being able speedily to cure it by the use of opium. I accordingly directed the following draught to be given at bed time.

℞. Liq. opii. sed. ♏xxx. Liq. ammoniæ acetat. ʒj. Mist. camph. ℥vij. fiat haustus.

15th. Slept well, and is this morning perfectly rational. On being informed of his delusion, he smiles, and appears to have some vague recollection of it. Says he has neither wife nor child, and cannot possibly conceive what brought such a notion into his mind. The nurse states that the fever came on as usual yesterday evening, and continued during the night; but his skin is now bedewed with perspiration, and his pulse soft and full, and down to 90. A marked change has taken place in his countenance, which is no longer either vacantly dull, or expressive of mental suffering.

Habeat quiniæ. sulph. gr. iij. quamprimum et repetat ter in die.

Haustus opiatus iterum sumatur h. s.

Persistat in usu pulv. alterativ.

After this date, it was not found necessary to repeat the draught, and the quinine agreeing well, the fever rapidly declined. On the 24th of January, the abdomen being flatulently distended and the seat of pinching pains, though not tender on pressure, a diarrhœa ensued, and several slimy evacuations were passed (the fluxus pituitosus of Hoffmann). The absence of abdominal tenderness, the negative state of the tongue, and the appearance of the slimy discharge (which consisted in a great measure of, apparently, mere mucus, without any of the albuminous matter which active irritation produces), induced me to give the copaiva, a medicine which I never prescribe unless in such an atonic state of the mucous membranes. I gave it in small doses, combined with *liquor potassæ* and laudanum, and its good effects were speedily visible. The quinine, omitted during its use, was resumed after a few days; and from that date the patient's recovery progressed uninterruptedly and rapidly. He was discharged, cured, on the 10th of February.

I have though it unnecessary to notice the variations in diet which were made from time to time, as the patient's state altered.

*Remarks.* It is not improbable that, in this case, the poison had remained latent in the system for a considerable time before the rain and cold, acting as an exciting cause, roused it into full action; for the disease seldom originates at so late a period of the year.

The patient's state on admission was far from being a promising one. Not only did he labour, with vital powers greatly reduced, under a fever which gave rise to inordinate action, and of which the remission was comparatively short; but he suffered also from an advanced stage of bronchitis, the bronchial tubes of both lungs being much obstructed with muco-purulent secretion. A careful consideration of all these circumstances furnished me with the following indications of cure: first to equalize the circulation, and thus (and, if necessary, by topical means also) to relieve existing congestions, particularly that of the lungs. Secondly, to improve the state of the secretions and excretions. Thirdly, to support, as need might be, the powers, avoiding all unnecessary stimulation.

To arrest the febrile paroxysms by the specific remedy, could be regarded only as an ulterior indication, consequent on the fulfilment of the other three.

It appeared to me that the agents best calculated to effect the primary objects above specified, were the tepid bath, alteratives, and aperients, together with such an application of leeches to the chest, as the strength of the patient might seem to justify. As an experimental essay on this point, I ordered six leeches to be applied on the evening of admission, and such was the marked efficacy of the bath in relieving the pulmonic congestion, that further leeching was not deemed necessary. Its effects on the circulation through the abdomen were scarcely less beneficial, as was testified in particular by the improved state of the epigastrium.

The ulterior part of the treatment has already been fully descanted on.

### INTERMITTENT FEVER.

The true cause of periodical intermittence in this and some other forms of disease, has hitherto eluded the discovery of all those who have investigated it. The older physicians indulged without restraint their fancy in the enquiry, and some French writers of our own day, as if to prove that folly and ingenuity had not yet exhausted themselves in attempting to solve the question, have advanced with regard to it doctrines and explanations, which, while they have the merit of being highly novel and ingenious, are at the same time, not only purely hypothetical, but also ridiculously absurd. (Vide Bailly, Roche, Brachet, &c. &c.) In the following observations of Werlhof (which I quote from the Dict. de Med. et Chir. Prat.) I am disposed fully to concur: "Typorum et periodorum febrilium miracula vidit omnis ætas, et obstupuit; videbit omnis posteritas, posteritas forsan omnis obstupescebit."

But though we are completely ignorant of the essential cause of intermittence and periodicity in disease, we can yet observe and reason upon the phenomena which accompany them. Thus if we consider carefully the symptoms presented by a whole paroxysm of intermittent fever, we shall have good grounds to infer:

First, that the primary link in the varied chain of morbid actions which constitute such paroxysm, consists in a particular impression on, or condition of, the nervous system.

Secondly, that the speedy result of this is a temporary diminution of power in the heart and vascular system generally, whence ensue retarded circulation, an imperfect propulsion of blood to the surface and extremities of the body, and a consequent congestion of it in the vessels and organs of the thorax and abdomen.*

Thirdly, that after this state has continued for a period *always shorter in proportion as the patient is more vigorous,* the heart and vascular system recover their power, and with forced action propel the blood to the circumference and extremities, giving rise to pyrexia, and subsequently (in a manner which we do not comprehend) to profuse cuticular secretion.

Fourthly, that when such paroxysms have recurred frequently, there is often produced either passive permanent distention, or active growth and enlargement, or, it may be, even inflammation, of those organs in which, from their *position, structure, vascularity, and peculiar circulation,* the congestion is greatest. I need hardly specify the lungs,† spleen and liver as the organs here chiefly alluded to.

---

* This appears to me a more rational exposition of the phenomena than that generally given, and which supposes an *active recession* of the vital fluid, from the surface and extremities to the internal organs.

† The congestion of the lungs in the cold stage of intermittent fever is noticed by the illustrious Harvey—sanguis in pulmones impingitur, incrassatur, non transit; hoc ex dissectione illorum qui in principio accessionis mortui sunt expertus loquor.—("*De Motu Sanguinis,*" *cap.* 16, *quoted from the Dict. de Med. et de Chir. Prat.)* Dr. Stokes, in the 31st vol. of the Ed. Med. and Surg. Jour-

After these, the pulmonary and gastro-intestinal mucous membranes (particularly the latter) are the parts which are most frequently and most extensively affected.

It is now pretty generally acknowledged, that cold and moisture are not of themselves sufficient to produce genuine intermittent fever, which owes its origin solely and exclusively to some endemic influence. Its prevalence in marshy situations, and during or immediately after certain states of the atmosphere, is a strong presumptive, though perhaps not an absolute proof, that vegetable decomposition is the source whence this influence originates. The fact so much insisted on by Dr. Good and others, that ague often prevails in elevated localities and in situations remote from marshy ground, proves nothing; inasmuch as we know that the circumstances which favour vegetable decomposition may exist on hills as readily, and indeed sometimes (as regards the proper *degree* of heat and moisture) even more readily than plains or valleys; while it is also certain that a very small quantity of vegetable matter undergoing decomposition in a locality otherwise healthy, may generate the disease. I once met with a case of tertian intermittent in the inmate of a cottage healthily situated, but near and before the door of which lay a few cabbage-stalks and leaves undergoing decomposition. The disease was rapidly cured by bark, the nuisance being removed; but recurred in the same patient, and presented itself also in another member of the family, some weeks afterwards, when another accumulation of the same substance, in precisely the same situation, had taken place. The supposed cause of the fever being now permanently removed, no case of ague presented itself in that family during the three subsequent years that they were within the reach of my observation. I recollect another case in which the disease was traced to a heap of willow twigs, thrown out by a neighbouring basket-maker into a drain which ran beneath the window of a low cottage chamber. All the members of the family who slept in that chamber (and from accidental circumstances it changed its occupants several times) were in turn affected with either remittent or intermittent fever—diseases previously unknown in that neighbourhood. Here too, on the removal of the nuisance, no case of ague ever afterwards occurred. I am always slow to admit, or believe in the *certainty* of any thing, but in both the instances here related, the origin of the disease appeared to me to be unquestionably ascertained.

That certain neuralgic affections originate in the same cause which produce intermittent fever, cannot well be doubted; for they prevail in the same localities with it, put on the same intermittent form, and are cured also by the same remedies. Whence then arise the marked differences observable between them? To regard, indeed, such affections as mere local diseases, would argue but a very imperfect acquaintance with the state of the general system in which they exist; but how comes it that in them the constitutional symptoms are, comparatively speaking, so faintly marked?—that the characteristic rigor and pyrexia are nearly or altogether wanting?—and, further, that an additional element is present, in the implication of part of the *sensitive* nervous system? These are questions the satisfactory solution of which must await future advances in pathological science. Almost all that we know upon the subject at present is, that the affections alluded to are produced, generally, by a long-continued exposure to the morbific cause; and that they are much more difficult of cure than simple intermittent fever. I have often been led to suspect that in them the organic system of nerves is more extensively affected, and that the implication of the sensitive system is only secondary; but these are merely points of belief, nal, mentions the frequent existence of this complication, as discovered by the stethoscope. Congestion of the liver and spleen is noticed by all who have written on the disease.

while the facts stated in the previous sentence are matters of positive observation. It would be well, I think, if writers on medicine were always to draw a clear line of distinction between what is *known*, and what is *merely believed*, to be.

The more external sensitive nerves are those most generally affected, possibly because all the larger ones run near the surface of the body, which both needs, and is endowed with a more exquisite sensation than the more protected parts of our frame. But the alimentary canal, *which is, as it were, another external surface*, is also sometimes the seat of intermittent neuralgic pain. I have met with many cases in which the stomach in particular was thus affected, and in which, after a very protracted duration, and the unavailing employment of a variety of remedies, the pain entirely ceased on the exhibition of quinine. In only a very small number of these cases was the pain regularly periodical, nor did it on intermission leave the stomach quite free from uneasiness; but its remissions and exacerbations, uninfluenced in a great measure either by food or abstinence, were sufficiently characteristic of its nature. In one case, in which the affection had existed for two years, the application of cupping-glasses to the epigastrium was followed by a distinct rigor, and the quinine being then given, the pain very speedily ceased to occur. All the patients thus affected had resided for many years in malarious districts, and the great majority of them were from one particular village. They were chiefly females. I shall again allude to these cases when I come to treat of affections of the stomach.

The duration of the *latent period* of intermittent fever is exceedingly uncertain, being apparently very much dependent upon the accidental application of an efficient exciting cause. In all the parts of Ireland with which I am acquainted, intermittent fever, as an indigenous disease, is altogether unknown; but it is occasionally met with among the numerous labourers who annually resort to England at the approach of harvest. In such patients I have known it to occur (generally during, or immediately after exposure to rain and cold) at periods varying from one to nine months after their return home. It is a remarkable fact that even in districts in which, for miles around, the whole surface of the country is covered with peat bogs, and of which many parts, during certain months of the year, are regularly overflowed with water—intermittent fever should never be produced. The preservative power of peat, and of the water which has been any considerable time in contact with it, is well known; and its influence in preventing vegetable decomposition must be very considerable; but how are we to account for the fact that in common meadow land (whatever may be the nature of the soil) intersected by streams which often overflow their banks, and leave stagnant pools behind them, genuine ague should never (I speak only of those parts of Ireland with which I am myself acquainted) be found to exist as an indigenous disease?

As regards the treatment of intermittent fever, I have nothing new to offer; notwithstanding I deem it not amiss to state my manner of procedure in the application of the old and commonly used remedies.

When a case of the disease comes before me, having enquired minutely into its type, duration, &c. I examine carefully the state of the abdominal organs, and, if need be, (which is comparatively rare) those of the chest also. If the disease be recent, and the epigastrium neither full nor tender, I begin by prescribing an emetic, which I repeat or not, as circumstances indicate. If, on the other hand, (which is much more commonly the case), the disease has existed for a considerable time, and the epigastric or either hypochondriac region is fuller than natural, cupping-glasses are ordered to be applied, and afterwards mercurial and saline purgatives are prescribed, until the hepatic and intestinal excretions are brought into a healthy state. As soon as the object is effected, the quinine is given.

When the spleen or liver is felt, or proved by percussion to be, enlarged; or

When, without such complication, the excretions show a strong tendency to become again disordered, I prescribe with the quinine five grains of blue-pill to be taken every night, or, in some cases, night and morning, administering also an efficient purgative once or twice a week.

The French practice of applying blisters over an enlarged spleen, and dressing the raw-surface with powdered digitalis, appears to me (from what I have witnessed of the effects of the endermic method generally) a highly rational proceeding; and I have tried it with success in a few instances, yet not often enough to speak decidedly as to its merits. I may take this opportunity of stating, that I have found the endermic method of administering remedies, *much more certain as regards the effects*, than the ordinary manner—as indeed might be expected after a just consideration of all the comparative circumstances—and I feel therefore persuaded that it must ere long come into general use.

When the abdominal complications above alluded to have existed for any considerable time, it not unfrequently happens that the patients become low-spirited, and indeed hypochondriacal, requiring not only to be encouraged by confident promises as to the ultimate result of their cases; but (what is of more importance still) to have all their present feelings and sufferings patiently attended to, and, if possible, relieved. By neglecting this important duty, medical men have sometimes incurred the unhappy reflection, that they did not do their utmost to lessen and remove that gloom and melancholy which ultimately led to suicide on the part of their patients.

Malignant or pestilential intermittent fevers (*les fievres intermittentes pernicieuses* of the French; the febres perniciosæ of Torti and other authors), were formerly not uncommon in this country,* and still prevail in many parts of the Continent, particularly in Italy. They occur in patients whose general functions have been deranged, and whose blood has been rendered impure by long-continued residence in an unhealthy malarious district; and it is to such a state of the system, and the congestions necessarily consequent upon it, that their malignity is mainly or, indeed, altogether attributable. This is the form of intermittent fever in which a combination of tartar-emetic and opium,† as recom-

* Vide Sir Geo. Baker, in 3rd vol. of Med. Trans.; Huxham in Operibus, passim, &c.

† In all diseases in which there is high general action, with a debilitated state of the vital organic powers, the blood is more or less diseased, and local congestions and inflammations are of very common occurrence. In such diseases an efficient yet cautious use of tartar-emetic alone will effect more good than any other remedy whatsoever. But if to the pathological conditions above-mentioned, there is added *an irregularly excited state* of the nervous system, the addition of opium to the tartar-emetic becomes necessary; and thus combined, opium is not only borne, but proves eminently beneficial under circumstances in which it could not with safety be given alone. This I can testify from having, during several years past, tried the combination in that form of typhoid fever accompanied with low muttering delirium, which so often supervenes upon pneumonia and other local inflammations, when they occur in debilitated subjects.

The profession is much indebted to Dr. Graves for the admirable manner in which he has proved and illustrated the efficacy of tartar-emetic and opium combined, in the treatment of delirium tremens, as well as of fever; but it has often appeared to me strange, that Dr. G., who is so well versed in German literature, is not aware of the fact, (and it is certain he is not, seeing that he has not mentioned it,) that in Rust's Magazine for 1831, there is an article by Dr. Thümmel, of Berlin, on the efficacy of tartar-emetic in pneumonia, croup, *and delirium tremens*. In this last disease he recommends the tartar-emetic *to be combined with an antispasmodic*, and the one he prefers is a strong infusion of mended by M. Peysson, is likely to prove most beneficial, whether given as a preparative for the bark, or as an accompaniment to its use.*

I treated altogether twelve cases of intermittent fever, ten of which were of the tertian, and two of the quartan type. Most of these cases had existed for a considerable period (vide tables) before admission, and were more or less complicated with local affections. A few were irregular in the recurrence of their paroxysms, and presented only slight chills, followed by partial flushings, and seldom terminating in perspiration. These were, in general, neglected or mismanaged cases of tertian fever, to which type they still shewed their affinity by an aggravation of their symptoms on alternate days, the intermediate days, however, being seldom quite free from their occurrence. But while, in this respect, they approximated to the nature of the quotidian, they affected, on the other hand, an approach to the quartan in the hour of their recurrence, which, though varying considerably, was generally late in the day. All of these cases had had a very protracted existence, and were, as might be expected, tedious of cure. The treatment of them was based on the following indications: first, to regulate and improve the general functions; secondly, to prevent the recurrence of the chills, &c. by the use of bark or quinine, combined generally with mild aperients. Sometimes the liquor arsenicalis was found useful, or (if the patient appeared anæmic) some of the preparations of iron, together with the bark; thirdly, to remove, by appropriate remedies, any local congestion or pain. In two cases, after equalizing the circulation and removing congestion by means of emetics and the application of cupping-glasses, the disease resumed its regular type, the several stages of each paroxysm became complete, and a perfect cure was then speedily established.

Some of the worst and most rapidly fatal cases of phthisis which have come under my notice during the period of my residence in Kent, have occurred in malarious districts, and have been complicated with *irregular* intermittent fever; a fact at variance with the notion of Dr. Wells, who fancied that wherever ague prevails, there consumption is very rarely, if ever, met with. It must not be

---

valerian or serpentary. In eight ounces of either of these he dissolves eight grains of tartar-emetic, and prescribes one table-spoonful of the solution to be taken every two hours. The medicine, he says, causes at first—vomiting of a bitter bilio-mucous liquid, then copious evacuations by stool, and, in the space of twelve hours, such a tranquil state of the system that the patient sleeps quietly and refreshingly for several hours together. By continuing the use of the remedy for two days, a perfect cure (vollhommne bèsserung) is, he assures us, effected. Two cases are related, and two others are referred to, as proving the truth of his statements.

Here then we have tartar emetic, combined with an antispasmodic, strongly, and on good grounds, recommended as a most efficient remedy in delirium tremens. How easy and natural the step from this to a combination of it with opium, the best of antispasmodics, and at the same time of tried efficacy against the disease in question! Far be it from me to insinuate that such was the process by which Dr. Graves was led to employ the remedy in the form recommended by him. My only object is to shew that the latter step in the discovery, as it was one of no difficulty, so ought it not to be regarded as of equal importance with the former. This will be admitted with peculiar readiness by those who think, with me, that the tartar-emetic *of itself* is a most invaluable remedy in the treatment of delirium tremens.

I may take this opportunity of stating further, that I have found this powerful and highly manageable medicine, whether alone or in combination with opium, as circumstances indicated, to be signally useful in the treatment of diabetes.

* The bark and tartar-emetic must not be given *in combination*, as the latter is decomposed by the former.

imagined, that I have mistaken the chills of phthisis for those of ague. This is an error which none but a very inexperienced person indeed could fall into. In such cases, I always found that the patient's state was rendered much more comfortable, and his general health slightly and temporarily relieved, by removing (as I have generally succeeded in doing) the aguish symptoms.

To illustrate more clearly the manner in which the several *local* complications that presented themselves in my aguish patients, were attended to, I shall give a succinct detail of seven cases (including the two of quartan fever) which I have selected for that purpose.

Case 1.—J. A. labourer, æt. 20, admitted May 6th.

*Symptoms.*—Experiences every morning about 8 o'clock a chill, which on alternate days lasts nearly an hour and an half, and is followed by pyrexia of about 4 hours' duration; after which a profuse perspiration breaks out over the whole body. On the intermediate days, the duration of the stages is considerably shorter, and the symptoms attendant upon them are much less severe. Conjunctivæ yellow; epigastrium painful, but neither full nor tender; bowels costive; tongue furred; pulse (towards decline of hot stage) 100, soft and full.

Disease commenced three months since, but in consequence of his taking pills given him by a non-professional person, has at different times ceased for periods varying from a few days to nearly a fortnight.

Pulv. ipecacuanhæ, gr. xxv. vespere.

May 7th. A lengthened paroxysm this morning, beginning at 8 o'clock.

Pil. hyd. c̄ col. gr. x. singulis noctibus.

Haustus Catharticus cras mane.

8th. No paroxysm. Medicine operated freely; evacuations dark, and offensive.

9th. A lengthened paroxysm this morning, beginning a little before 8 o'clock.

Pulv. ipecacuanhæ, gr. xxv. vespere.

Cras mane, sumat Pulv. quiniæ sulph. gr. iij. et repetat ter in die.

10th. A milder paroxysm this morning, beginning exactly at 8 o'clock. Epigastrium now feels full.

C. C. c̄ ferro, epigastrio. Persistat in usu quiniæ sulph.

After this date no paroxysm occurred; but on the 16th, the stomach being disordered, with foul tongue, another emetic was given. On the 20th the patient was discharged cured, with directions to return to the hospital in case the disease recurred; but he did not again present himself.

*Remarks.*—This was a case of what is called "double tertain," there being a paroxysm every day, but those on alternate days only corresponding in duration and severity. In its recurrence at so early an hour in the morning, the disease approximated to the character of the true quotidian, the existence of which as a distinct type is questioned by some authors; and is undoubtedly very rare. The stomach, in the present case, was much disordered, but greatly relieved by the emetics. After that given on the 7th, a day passed without any paroxysm whatever. This effect I have often seen to follow the employment of an emetic. As soon as the epigastrium was felt to be full, cupping-glasses were applied; and, the fulness subsiding, I did not afterwards hesitate to prescribe another emetic. With the exception of the nights on which the emetics were administered, the purging pills were given every night during the patient's stay in the hospital.

Case 2.—S. C. an unmarried female, æt. 22, admitted May 20.

*Symptoms.*—Early in the afternoon of alternate days, she experiences a sensation of chilliness, which, lasting from half an hour to nearly an hour, is succeeded by flushes of heat, generally partial, and continuing for a space of between two and three hours. After this, partial perspirations appear on different parts of the body. General health much impaired; countenance sallow; temples affected with a severe throbbing intermittent pain, which occurs at irregular periods; dull aching pain of left side; epigastrium soft; tongue furred; bowels costive; pulse (during intermission) 82, feeble; catamenia profuse, occurring usually after an interval of scarcely a fortnight, and continuing during the greater part of a week.

Disease has existed five months.

*Treatment.*—For six days after admission, the patient took, three times in the twenty-four hours, a mixture containing sulphate of magnesia, infusion of senna, and infusion of quassia. This brought away daily, two or three stools, at first dark and offensive, afterwards highly charged with bile. On the 26th, two grains of quinine were ordered to be taken three times a day, the mixture being still continued. On the 28th, the mixture was omitted, and ten grains of alterative powder prescribed to be taken every night. On the 30th, the pain in the temples being very severe, an emetic was administered: it operated freely, and from that moment the pain entirely ceased. On the 4th of June, irregularly occurring chills being still occasionally felt, five drops of liquor arsenicalis were ordered to be taken in distilled water three times a day, the quinine being still continued. On the 13th of June, a blister was applied to left side, which still continued at times painful. The stomach feeling uneasy, the liquor arsenicalis was omitted on the 20th of June, and the patient was discharged cured on the first of July, the general health being then quite re-established.

*Remarks.*—The digestive organs being very much out of order, I, for some days, confined my attention in a great measure to them, prescribing, in small and frequently-repeated doses, purgatives combined with a bitter—a mode of treating chronic disorders very common among the older physicians, and well deserving of imitation. Even after the use of the quinine was commenced, I deemed it advisable to continue a little longer the purgatives; and on omitting them, two days afterwards, a full dose of the alterative powder was ordered to be taken every night. It is worthy of observation that after the bowels were thoroughly cleansed, and brought to act regularly and efficiently, the catamenial discharge became less frequent, and less profuse; and this amendment had continued when I heard of the patient six months after she had left the hospital. I have often indeed found that a menorrhagia which had resisted all the usual remedies, has yielded speedily on purging the patients well with salts and infusion of roses. When this symptom exists independently of any discoverable uterine cause, the intestinal secretions are, I believe, very generally depraved, the large intestines being at the same time loaded with fæces.

The effect of the emetic in relieving the pain of the temples was both speedy and decided.

The pain in the left side being sympathetic, must have ultimately yielded to the general treatment; but as the patient often complained of it, and as it was possible the spleen, though not enlarged so as to be felt, might yet be the seat of congestion, a blister was applied, and the pain in consequence altogether ceased.

It is chiefly in *irregular* ague that the liquor arsenicalis is found to be serviceable. I prescribed it in the present case for rather more than a fortnight, but discontinued it the moment that gastric uneasiness was complained of. I never trust to it alone, but prescribe with it either the bark or quinine, even in those cases in which one or other of these has already been repeatedly given without affecting more than a temporary cessation of the paroxysms.

CASE 3.—O. W. labourer, æt. 19, admitted June 3rd.

*Symptoms.*—Chilliness, followed by flushes of heat and partial perspirations, occurring daily between twelve and one o'clock, but severer and of longer duration on alternate days; dull continued pain of head; countenance pale and slightly bloated; skin of a waxy appearance; action of heart much increased and irregular; epigastrium soft; tongue clean; bowels costive; pulse 90; feeble.

Disease has existed five months.

*Treatment.*—Pulv. ipecac. ℈j. vespere, et postea pediluvium.

Haust. catharticus cras mane.

June 6th. A paroxysm each day since admission, those on alternate days corresponding. Bowels now regular in their action.
Capiat ter de die, pulv. cinchon. rubr. ʒj.
Singulis noctibus, sumat pil. hyd. c̄ col. gr. v. vel. gr. x.

8th. Throbbing pain of head.

Vomitorium vespere.

After this the head remained free from pain, but the paroxysms did not altogether cease till the 12th, when the patient's general health began rapidly to improve. On the 22nd he seemed fit to be discharged, but on the following day he complained of pain in the right groin, the glands of which were found to be enlarged and very tender, requiring the application of leeches. He subsequently suffered from severe pains in the loins; and his heart, which had for some time been quiet, began again to be tumultuous and irregular in its action. On the 7th of July, he had a convulsive fit, resembling that of epilepsy, but without foaming at the mouth. On its cessation he remained for several hours in a soporose state. Cupping-glasses being applied to the region of the heart, its action became more tranquil and regular; and, the fit not returning, the patient was discharged cured on the 22nd of July.

*Remarks.*—This also was a case of *double tertian,* which yielded without much difficulty to the remedies employed. The patient's stay in hospital was much protracted by the superinduced affections of the groin and loins, as well as by the recurring irregular action of heart, and the convulsive fit consequent thereupon. The patient, on being strictly questioned, acknowledged the correctness of a suspicion which I had for some weeks entertained—that he was addicted to a disgusting and baneful practice.

Case 4.—J. C., labourer, æt. 29, admitted August 12.

*Symptoms.*—Countenance heavy and vacant; pupils contracted; acute and constant pain in head; nausea and occasional vomiting; pulse 90, sharp; tongue furred; bowels open; urine high-coloured and scanty.

Rather more than a fortnight since, while working, with his head uncovered, under an intensely hot sun, experienced suddenly, nausea with a sense of great weakness, and a few minutes afterwards was attacked with a convulsive fit, resembling, according to his own account, that of epilepsy. This has since frequently recurred, lasting generally about half an hour. On the morning after the first attack, he was seized with a rigor followed by heat and perspiration, which has since several times returned, but he cannot state whether at regular or irregular intervals.

*Treatment.*—Abradatur capillitium, et, caloie preter modum aucto, adhibeantur capiti lintea in aqua frigida immersa.
Ext. colocynth. comp. gr. v. Hyd. submur. gr. v. fnt. pil. ij. h. s. sumendæ.
Haust. cath. cras mane.

These remedies were repeated on the 13th, and again on the 14th.

16th. Since admission, has had an ague-fit every second day, the accession varying from eleven to one o'clock. Head much relieved by purgatives. Epigastrium tender and full, but not tense.
C. C. c̄ ferro, epigastrio.
Pil. hyd. gr. v. singulis noctibus.

18th. No rigor; pain of head quite gone; countenance more animated; pulse 82, soft; bowels open; tongue moist; urine deposits a copious sediment.

20th. No return of rigor or head-ache. Pulse feeble.

Sumat bis die quiniæ sulph. gr. v.

22nd. Epigastrium still full and tender.

Applicentur C. C. c̄ ferro. Persistat in usu quiniæ sulph.

The improvement from this date was rapid, and the patient was discharged cured on the 3rd of September.

*Remarks.*—In this case, the *exciting* cause of the intermittent fever was, in all probability, the *coup de soleil* to which the patient was exposed on the day previous to its first accession. The head-affection claimed of course my first care, and the means resorted to were found sufficient to subdue it without having recourse to blood-leeting. It is worthy of remark that, in consequence of the cupping and purgation, the paroxysms had ceased during a period of four days before the quinine was given; but they would undoubtedly have recurred had the specific remedy not been resorted to. I have again and again succeeded by the removal of local congestions, in stopping for a time the paroxysms, but, the specific being withheld, they have invariably, after a longer or shorter interval, returned.

CASE 5.—W. W., labourer, æt. 41, admitted April 11, 1837.

This was a case of tertian ague complicated with bronchial catarrh, both induced, three weeks previously to admission, by the patient's sleeping in a damp bed. His general health was much impaired, the countenance being sallow, the skin harsh, and the pulse feeble. There was troublesome cough with profuse expectoration of a thin albuminous-looking matter.

*Treatment.*—This for the first three days consisted of an emetic, a blister to the chest, purgatives, and an opiate in combination with blue-pill each night at bed-time. On the 14th, the quinine was administered, and from this date the patient's recovery progressed daily. He was discharged cured on the 2nd of May.

CASE 6.—J. D. æt. 13, admitted December 2nd.

*Symptoms.*—A chill, followed by heat and perspiration, recurring every third day, about 5 o'clock, p. m., the paroxysm lasting till 7 or 8 o'clock the following morning. Countenance sallow and bloated; lower extremities and scrotum œdematous; abdomen soft; tongue clean; bowels regular; pulse 110, feeble.

Disease began as a tertian intermittent about five months ago, but has for more than half that time presented the quartan type.

*Treatment.*—Pulv. ipecac. gr. xv. vespere.

Haust. catharticus cras mane, nisi vomitorium inferiorem ventrem expurgaverit.

Purgatione finita, sumat quiniæ sulph. gr. ij. 6ta q. q. hora.

Only two paroxysms occurred after admission, namely, one on the third, and the other on the 6th. In addition to the quinine powders, I prescribed, on the 8th, small doses of the tinct. ferri. mur. in infusion of quassia, also five grains of the alterative powder, to be taken every night at bed-time. By means of these remedies, and a generous diet, the patient's strength increased daily, the œdematous state of the lower extremities and scrotum gradually disappearing. He was discharged cured on the 23d, and was to have left the hospital on the following day; but the roads being obstructed with snow, his disappointment at the delay brought on headache and great depression of spirits, which rendered a temporary suspension of his tonic medicines advisable. They were resumed on the 31st, and he returned home on the 13th of January, his health being perfectly re-established.

Case 7. E. G., a married female, æt. 34, admitted an out-patient Dec. 2nd.

*Symptoms.*—General chill, succeeded by flushes of heat and perspiration, recurring every third evening at 7 o'clock, the paroxysm lasting until about 9 on the following morning. Complexion of a dusky sallow hue; lower extremities anasarcous; abdomen contains fluid; bowels costive; excretions unhealthy; tongue clean; appetite impaired.

Three years and a half ago became the subject of tertian fever, which at first was very imperfectly treated. It continued to affect her at times until May last, when, as an out-patient at the hospital, under my care, she was apparently cured, and remained indeed quite well until about two months since, when the disease again shewed itself, but under the quartan type. Anasarca of lower extremities and swelling of abdomen have existed four weeks.

*Treatment.*—Pil. scillæ co., Pil. hydrarg. āā ʒj. M. fnt. pilulæ xxiv. sumat duas singulis noctibus et unam singulis matutinis.

Pulv. cinchon. ʒj. pot. nit. gr. x. ft. pulvis, 6ta. qq. hora sumendus.

Dec. 16th. Mouth became affected four days ago, since which no paroxysm. General health improved. Pills have been omitted.

Persistat in usu pulv. cinchonæ, et potassæ nitratis.

23d. Mouth now nearly well. Paroxysms have not returned. Anasarca of lower extremities has completely disappeared, and abdomen no longer fluctuates. General health much improved; complexion has in a great measure lost its dusky hue; tongue clean; bowels regular; appetite good.

She was discharged cured on the 30th of the same month.

*Remarks.*—Was the disease in this case the mere posthumous offspring (if I may use such an expression) of the former long-existing tertian? or was it an entirely new production, the result of a fresh application of the morbific poison? I am inclined to look upon it in the latter light, both on account of the long period intervening between the cure (to all appearance perfect) of the tertian, and also because of the last attack occurring at a season of the year so favourable to the production of original quartans. But the fact is one which cannot, I think, be unquestionably determined.

The anasarcous condition of the lower extremities, the fluctuation felt in the peritoneal cavity, the state of the excretions, the dusky sallow hue of the skin—all convinced me that the liver was to a considerable extent implicated. I prescribed therefore the blue-pill combined with a diuretic; and as the patient's strength was much reduced, and there was neither headache nor heat of skin, I lost no time in commencing the use of the bark, which was given in combination with nitre, a very old formula, and one which appeared to me well adapted to the present case. As soon as the mouth became affected (and not till then) the paroxysms ceased. From the same moment the anasarca began to decline, the complexion to become clear, and the general health to improve; and the patient, when discharged (four weeks after her admission), appeared, as it were, a totally different person.

### WESTERN LYING-IN HOSPITAL AND DISPENSARY,
### (*Arran-Quay, Dublin.*)

### MEDICAL REPORT BY FLEETWOOD CHURCHILL, M.D., PHYSICIAN ACCOUCHEUR.

Dr. Churchill has forwarded to us two Reports—one for the year comprised between the dates Oct. 31, 1835, and Nov. 1, 1836—and a second for the period extending from Nov. 1, 1836, to Dec. 31, 1837.

We fear that our limited space will not permit us to insert both Reports in full, but we shall feel great pleasure in laying the second before our readers, and we shall feel equal pleasure in presenting condensed reports of the practice of Dr. Churchill in this lying-in institution on future occasions. There is no department of our science which merits more attention than obstetrical, none possessed of more practical importance, none involving higher or more interesting questions.

It appears that the Western Lying-in Hospital and Dispensary was established in the Autumn of 1835, for the purpose of receiving, or attending at their own homes, the poor females of the western parishes.

It was set on foot mainly by the clergymen of these parishes, and by them, in conjunction with several laymen and a committee of ladies, it is managed.

It is supported by donations, subscriptions, and the collections made after charity sermons in the parochial churches, in addition to the fees paid by pupils, the right to which has been made over to the committee by the medical officers, for the purpose of perfecting and extending the hospital accommodations.

Between Nov. 1, 1835, and Oct. 31, 1836, 247 women were relieved—of these 97 were received into the hospital, and 150 attended at their own homes. There were eleven cases of abortion, leaving 236 cases of labour, and these 236 cases produced 240 children, there having been four cases of twins.

Having thus explained the nature of the institution, and the number of patients treated in the first year, we shall insert Dr. Churchill's Report for the second year.

This Report will include a period of fourteen months, *i. e.* between Nov. 1, 1836, and December 31, 1837.

During this period 391 females have derived assistance from the hospital: of these 128 were intern and 263 extern patients. From this number we must deduct 21 cases of abortion and one case of uterine hydatids, which will leave 269 cases of labor at the full term of utero-gestation.

The number of children born, amounted to 376, of which 206 were males and 170 females. 28 of them (17 male and 11 female) were still-born, or died immediately after death; of these 28,

| | | |
|---|---|---|
| 5 were premature. | | 2 were funis presentations. |
| 2 — breech presentations. | | 1 was a footling case with prolapsed funis. |
| 2 — footling presentations. | | 1 was covered with syphilitic eruption. |

We obtained the ages of 313 as accurately as possible, there were

| | |
|---|---|
| 21 cases under 20 years | 42 cases between 30 and 35 |
| 97 — between 20 and 25 | 42 — — 35 — 40 |
| 106 — — 25 — 30 | 2 — — 40 — 50 |
| | 313 |

It is not always possible to ascertain the exact commencement of labor, and where any cases were doubtful, they have been omitted in the registry. For this reason I can only give the duration of labor in 299 cases instead of 369.

| | | | |
|---|---|---|---|
| In 65 cases it was under 6 hours. | | In 7 cases it was under 48 hours. | |
| 93 — — | 12 — | 5 — — | 60 — |
| 105 — — | 24 — | 5 — — | 96 — |
| 18 — — | 36 — | 1 — — | 120 — |

It may be as well to mention that the great length of time some of the labors were allowed to continue, did not arise from the neglect of the medical officers, but from the patient's deferring so long her application for assistance.

The interval between the setting in of true labor-pains and the rupture of the membranes has been carefully noted in 271 cases.

| | | | |
|---|---|---|---|
| In 60 cases it was about 2 hours. | | In 3 cases it was under 30 hours. | |
| 58 — — | 6 — | 6 — — | 35 — |
| 41 — — | 10 — | 1 — — | 40 — |
| 47 — — | 14 — | 4 — — | 50 — |
| 20 — — | 18 — | 1 — — | 60 — |
| 12 — — | 22 — | 3 — — | 80 — |
| 14 — — | 26 — | 1 — — | 108 — |
| | | 271 | |

In the same number of cases, the interval between the rupture of the membranes and the birth of the child was as follows:—

| | | | |
|---|---|---|---|
| Under 1 hour in 136 cases. | | About 15 hours in 9 cases. | |
| About 2 — | 40 — | — 20 — | 3 — |
| — 4 — | 46 — | — 25 — | 5 — |
| — 6 — | 19 — | — 35 — | 1 — |
| — 8 — | 7 — | — 40 — | 1 — |
| — 10 — | 3 — | — 50 — | 1 — |
| | | | 271 |

From the birth of the child to the expulsion of the placenta, there elapsed

| | | | |
|---|---|---|---|
| In 69 cases | 5 minutes. | In 3 cases | 50 minutes. |
| — 54 — | 10 — | — 11 — | 60 — |
| — 63 — | 15 — | — 15 | from 1 to 2 hours. |
| — 39 — | 20 — | — 2 | — 2 — 3 — |
| — 3 — | 25 — | — 2 | — 3 — 4 — |
| — 31 — | 30 — | — 4 | — 5 — |
| — 7 — | 35 — | — 1 | — 6 — |
| — 9 — | 40 — | — 1 | — 8 — |
| | | 314 | |

The cases in which so long an interval occurred between the expulsion of the child and the placenta had all been managed by midwives, who made application for assistance on account of the retention of the placenta.

In 358 cases the presentation was as follows:

In 331 it was natural.
— 4 the hand descended along with the head.
— 9 the breech presented.
— 10 the feet—in three of which the funis prolapsed.
— 2 the funis presented.
— 2 the arm.

358

I have already mentioned that of the breech cases 2 were lost:—of the footling, 3:—of the funis 2:—of the arm presentations one child was extracted alive, and the other was still born.

There were 7 cases of twins, their sexes were

| | | | |
|---|---|---|---|
| In the 1st case | | 2 males. | |
| — 2nd — | | 2 — | |
| — 3rd — | | 1 — | 1 female. |
| — 4th — | | 2 — | 0 — |
| — 5th — | | 1 — | 1 — |
| — 6th — | (premature) | 0 — | 2 — |
| — 7th — | | 2 — | 0 — |
| | | 10 males. | 4 females. |

Of these one male and two females (premature births) were still-born or died immediately after birth.

As to the presentations in these cases:—in four cases both the children presented naturally, and one child was lost;—in the fifth case, one child presented the breech and the other an arm;—in the sixth case, one child presented naturally, and the other was a breech presentation; in these two last cases all the children were saved;—in the seventh case, the presentation of one child was natural, and the hand of the other descended along with its head; both children were lost, as the labor was premature. In five cases hæmorrhage occurred before the expulsion of the placenta; and in four of them, that organ had to be extracted. The flooding produced no unfavorable results.

One patient was threatened with convulsions, but by the timely employment of antiphlogistic measures, she escaped.

In three cases, version was practised with considerable success. All the mothers recovered, and one child was saved.

The forceps were once successfully applied, both mother and child recovered.

In one case, the use of the perforator was required from the narrowness of the pelvis—the patient recovered.

Out of 391 females, three only died, and two at least of these, who were externs, were indebted rather to the mismanagement of friends, than to any accident of labor, for the fatal result. The third sank from the effects of the shock of a tedious labor upon a constitution debilitated by severe pulmonary disease. These cases will be related in a subsequent part of this report.

During the past year, we have had no cases of puerperal fever among either the extern or intern patients. In a few cases, the patients were attacked with intestinal irritation, or threatened with hysteritis, but by judicious and prompt treatment the symptoms subsided. In one case these symptoms were more severe, and terminated in the evacuation of a pint of purulent matter from the uterus "*per vaginam.*"

The measurement of the navel-string has been continued, but as the results only confirm the opinions advanced in a paper "On the Length of the Cord, &c." which I published in the Dublin Journal for March, 1837, the details are here omitted.

So far I have confined myself to a statement of facts taken from the hospital registry, but it may be useful to enter into somewhat more detail upon some of the cases and observations.

During more than half of this last year, two very intelligent pupils of mine (Mr. Gray and Mr. Gibbon) kept registries of several important points in the history of natural labor, concerning which some statistical information seemed desirable. We endeavoured, by taking down a number of cases, to ascertain the effects of the nervous shock upon females of different temperament after labor of varying duration. The pulse was examined just before delivery and at short intervals afterwards, and its alternations noted. The changes in the color, consistence, and quantity of the lochial discharge, were observed. The gradual and regular contraction of the uterus was remarked, and the period of the secretion of milk entered in the registry. By comparing these observations we were enabled, not only to ascertain with considerable accuracy the progress of convalescence after natural labor with the normal succession of phenomena, but were also made acquainted with certain deviations from the natural order, which were of some importance, though involving no organic lesion. The results of their registries, and of our observations, were published in the Dublin Journal for September, 1837.

*Tedious Labor.* In the report of the hospital for 1835-6, I took occasion to allude to the influence which the length of the first stage has been supposed to exercise upon the entire process, and upon the well-being of mother and child. Taking the rupture of the membranes as the limit of the first stage, (which is generally though not always the case,) and referring to the tabular view I then gave of the intervals from the commencement of labor to the rupture of the membranes, and from the rupture of the membranes to the expulsion of the child, I observed—

"1st. That the length of the period after the evacuation of the liquor amnii bore no proportion to the time which elapsed previously;" and—

"2dly. That the constitutional effects of labor are not to be estimated by the length of the first stage," &c. &c.

In order to bring these opinions to the strict test of statistical enquiry, I have this year taken (but not selected) 21 cases of tedious labor, the duration of which was 36 hours and upwards, and the details of which are minutely noted in the registry, and I have marked the duration of the 1st and 2nd stages of labor in each case respectively, with the results of the labor to the mother and the child.

1*st Group*—9 *Cases of Labor, of* 36 *hours' duration.*

| No. of Cases. | Length of 1st Stage. | Length of 2d Stage. | Results to Mother. | Results to Child. |
|---|---|---|---|---|
| In 5 cases | 35 hours | 1 hour | Favorable | Favorable |
| 2 do. | 34 do. | 2 do. | do. | do. |
| 1 (premat.) | 32 do. | 4 do. | do. | Dead. |
| 1 case | 25 do. | 11 do. | do. | Favorable |

2*d Group*—4 *Cases of Labor lasting* 48 *hours.*

| No. of Cases. | Length of 1st Stage. | Length of 2d Stage. | Results to Mother. | Results to Child. |
|---|---|---|---|---|
| In 1 case | 47 hours | 1 hour | Favorable | Dead—funis presentation |
| 1 do. | 47 do. | 1 do. | do. | Favorable |
| 2 do. | 45 do. | 3 do. | do. | do. |

3*d Group*—6 *Cases of Labor of* 60 *hours.*

| No. of Cases. | 1st Stage. | 2d Stage. | Results to Mother. | Results to Child. |
|---|---|---|---|---|
| In 3 cases | 59 hours | 1 hour | Favorable | Favorable. |
| 1 do. | 57 do. | 3 do. | do. | do. |
| 1 do. | 53 do. | 7 do. | do. | do. |
| 1 do. | 39 do. | 21 do. | do. | do. |

*4th Group—3 Cases of 96 hours.*

| No. of Cases. | 1st Stage. | 2d Stage. | Results to Mother. | Results to Child. |
|---|---|---|---|---|
| In 1 case | 95 hours | 1 hour | Favorable | Favorable |
| 1 do. | 93 do. | 3 do. | do. | do. |
| 1 do. | 90 do. | 6 do. | do. | do. |

The importance of this small series of well-authenticated facts will be at once perceived, and its bearing upon the recent controversy between Dr. Hamilton of Edinburgh, and Dr. Collins of this city, on the consequences of allowing the first stage of labor to exceed a certain time. As far as they go, they are diametrically opposed to the conclusions put forth by the former distinguished author. In none of these cases did the extraordinary length of the first stage "render the powers of the uterus inadequate to expel the infant with safety to its life or to the future health of the patient;" for out of 21 cases of tedious labor in the 1st stage, in only one instance did the 2nd stage exceed eleven hours; in 17 it was completed in three hours: none of the mothers were injured, and only two children lost, one of which was premature, and the other a funis presentation, and both deaths consequently independent of the duration of the labors. Neither did the secondary evils, "retention of the placenta, fatal hæmorrhage, or febrile and inflammatory affections," follow such prolonged first stage, for every one of the women recovered well. Then I would conclude that the duration of the second stage, and the results of the entire labor, have no relation to the length of the 1st stage, and further, we have at least negative evidence that it is upon the 2nd stage that the character of the labor and its result depends, for out of 21 very tedious labors, there was not one tedious convalescence—but then in all but one the second stage was short.

The *causes* of delay were such as are enumerated in standard works on the subject—premature evacuation of the liquor amnii—rigidity of the soft parts—depression of the anterior lip of the os uteri (*Hamilton*), &c.; and the treatment was in accordance with the recommendation of writers of authority.

One of three fatal cases, was a case of tedious labor. At the same time, in accounting for her death, it must not be forgotten that she had suffered from a severe pulmonary complaint before and during parturition. She was admitted into hospital on the evening of the 8th of April, 1837. She had some pains, which completed the first stage in 26 hours, without the slightest exhaustion or acceleration of pulse. The progress of the head, after it entered the pelvis, was slow though perceptible for the next twelve hours, but as the 2nd stage was so prolonged, it was deemed advisable to call in the consulting accoucheur (Dr. Darley), who gave it as his opinion, that the patient's strength was adequate to the delivery without assistance. In this he was right, for in two hours afterwards a male child was born, and the after-birth was expelled in half an hour. The pulse immediately after delivery was about 120, and she seemed much exhausted by the length of the labor and by her cough. She sank on the third day without any very marked symptoms. We could not obtain a necroscopic examination.

*Version Cases.* It was found necessary to extract the child by the feet in three cases. In the *first* case the arm presented with a loop of the funis. The waters had been discharged an hour and a half before assistance was obtained, and the uterus was acting briskly. Mr. Speedy succeeded, with some difficulty, in extracting the child, which was still-born. The woman recovered well. The *second* case was one of twins; the first child presented naturally, and was born after a short labor, but on making a vaginal examination, Mr. Speedy found a second, presenting an arm; he turned the child instantly, and succeeded in extricating it alive. The operation was facilitated by the previous transmission of the first child through the passages. Both the children with their mother did well.

In the third case I operated myself. It was a funis presentation, admitted into the Hospital June 12, 1837. The waters were evacuated about 3 p.m. and then the prolapsed end was discovered. On my arrival at 4 p. m. I found the funis still pulsating, and as the woman seemed well formed, I determined to attempt to save the child by turning. The extraction was executed with sufficient rapidity, until the head arrived at the upper strait, but so much delay then took place that the child was lost. The woman recovered.

*Forceps Case.*—But one forceps case occurred out of 391 patients; it is entered in the case book as follows:—M. Broderick, æt. 28, was taken in labor of her first child at 2 p.m. June 28, 1837. The midwife in attendance sent for the assistant-surgeon (Mr. Speedy,) of the hospital, to pass the catheter at 5 p. m. June 29. Upon inquiry, he found that the "waters" had been discharged at 3 a.m. that morning, and that the head shortly afterwards descended into the pelvis. A pint of high-coloured urine was drawn off, and a purgative enema administered. At nine o'clock the same evening a second visit was paid, but no advance of the head had taken place, so that it had remained in the same situation for at least 12 hours.

The pains had diminished in force and frequency, she was a good deal exhausted, her pulse 120 and weak, skin hot and dry, tongue white, great thirst. A consultation was obtained immediately, and no hesitation was felt in recommending instant delivery. As there appeared no want of the necessary space in the pelvis, it was determined to apply the forceps, and after a short delay, Mr. Speedy succeeded in extracting a living child. The placenta was expelled in 20 minutes. Both mother and child recovered well.

*Crotchet Case.*—Perforation of the child's head and extraction was performed once under very unfavourable circumstances. The patient, Mary Corr, æt. 30, had suffered from ill-health during the latter months of pregnancy. She was taken in labor on the 7th of Sept. 1837, the pains continued for two days moderate, and were gradually increasing in strength, when they were suspended in consequence of a quarrel with her husband. She remained for many hours with but very feeble pains, and probably without any real progress.

On the 12th of Sept. 1837, at 7 a.m., Mr. Speedy was sent for, and he found the head presenting and impacted in the upper strait. The 'waters' had not then been discharged, the bowels were constipated and the urine retained. The pulse were very quick and the skin hot and dry. The catheter was passed and a quart of fætid dark brown urine drawn off, a purgative enema was given and the membranes ruptured. The uterine action revived and strong pains recurred every five minutes.

In the evening a consultation was held, and as all the constitutional symptoms were aggravated without the least advance of the head, it was thought hazardous to defer the delivery any longer. The head was lessened and the child extracted in half an hour, followed shortly after by the placenta. The woman recovered without a bad symptom. The history of the early part of this case is as indistinct as the management was mischievous, and owing to the same cause, viz. the stupidity of her female attendants and the drunkenness of her husband.

She was almost delirious from long-continued pain and the incessant teasing of the women, and considering that the operation was performed in a wretched cellar, great inconvenience, and under sundry threats of bodily chastisement from the drunken husband and his fellows, it is a matter of surprise that the case was so successful.

Of the cases of *retained placenta*, two were worthy of notice. The first occurred after a premature labor, and it was found impossible to remove the placenta, although it was very desirable to have done so, on account of a considerable loss of blood. We had recourse to the plug which effectually restrained the hemorrhage and enabled us to wait the expulsive action of the uterus. This was not excited till 60 hours after the birth of the child. The woman suffered a good deal from irritative fever, but by strict regimen and antiphlogistic treatment, the patient gradually recovered.

The second case had a less favourable issue, though her labor terminated quite well. She was delivered of twins, in the 7th month of her pregnancy under the care of a midwife. The first child was born after a labour of 24 hours, duration, at 9 a.m., August 10, 1837; and the second at noon on the same day. The first presented with the head, and the second with the head and hand.

Between the birth of the children considerable hemorrhage took place, but it was not until some time afterwards that the midwife sent for assistance. Mr. Speedy found the patient greatly reduced, the surface of the body cold and exsanguined, the pulse almost imperceptible, &c.

As the after-births were still retained, he introduced his hand and removed them, having previously administered a cordial. Two days after, the abdomen became painful and tender and the pulse accelerated. Some ounces of blood were taken, and calomel with opium prescribed. This treatment succeeded and the patient became convalescent on the 17th. That very evening she got out of bed, quarrelled and fought with some lodgers, and took a quantity of whiskey and water. In the course of the night she was attacked with rigors, abdominal pain, &c. and the next day presented symptoms of abdominal inflammation with great sinking. In spite of our most diligent efforts she sank during the second night from the last seizure. We obtained permission to examine the abdominal viscera, but after the most diligent search could discover no morbid appearance. There was neither peritonitis, enteritis, hysteritis, nor rupture, so that we learned nothing as to the immediate cause of death.

With regard to the third fatal case—I may add a few words.—She was delivered safely of an acephalous fœtus and was progressing satisfactorily until some imprudent person shewed the child to her. She was taken ill immediately afterwards, and died with symptoms of intestinal inflammation. No '*post-mortem*' could be obtained.

I shall only add one more case—that of uterine hydatids. Ann Curwen, æt. 27, the mother of two children, and generally enjoying good health, menstruated regularly up to the end of August, 1836—the menses ceased after that time, from pregnancy as she believed; about a month afterwards she observed a slight discharge from the vagina, resembling blood and water, which continued three months or more, up to December 18, 1836, when she was attacked with labour pains, and all the signs of abortion, except that, instead of an ovum, a large basin-full of hydatids was expelled with considerable hemorrhage. She recovered perfectly under the ordinary treatment.

---

### CITY OF LIMERICK INFIRMARY.

### INJURIES OF THE KNEE-JOINT.

#### 1. COMPOUND DISLOCATION OF THE KNEE-JOINT.

James Tracey, a labourer, 50 years of age, was admitted to Barrington's Hospital, July 19th, 1836, at 9 o'clock, A. M. He was previously employed taking down part of an old building, on one of the walls of which he was standing, the wall gave way, his right leg was caught between the stones, and he stated that another part of the wall fell on his right thigh which caused him to fall forcibly forwards. He was immediately carried to the Hospital, when great deformity and shortening of the limb was evident. On examination the external condyle of the femur was found protruded downwards, outwards and backwards, through a wound of nearly two inches in extent—the patella and head of the tibia were thrown upwards, inwards, and forwards. There was some bleeding and escape of synovia from the wound. A lacerated wound of the same leg, which laid bare the bone and divided the fascia and muscles, extended nearly from the head of the tibia to the inner malleolus. The dislocation was easily reduced—the limb laid on a long splint in the straight position—and cold evaporating lotions ordered—the wound having been lightly dressed—a consultation was summoned and amputation proposed as soon as reaction was fairly established.

At this time there was little pain or swelling of the limb, and the patient obstinately refused to submit to amputation; under these circumstances I ordered 24 leeches to be applied to the joint, the use of evaporating lotions afterwards, and a mild aperient.

The subsequent details must be compressed. On the 21st, erysipelas made its appearance over the internal malleolus. This was subsequently checked by the application of the nitrate of silver solution. Synovia escaped at intervals from the joint. On the 24th he had a rigor. On the 26th pus escaped from the joint. On the 3rd of August, an abscess was opened on the outside of the knee-joint. The patient again refused amputation. On the 7th, another abscess was opened on the inside of the knee. On the 8th, a considerable arterial hæmorrhage took place from the joint. The tourniquet was applied, and amputation again recommended, and again refused. The limb grew black, and, at 6 A. M. next day, the patient died.

*Dissection.*—The leg appeared to be connected with the thigh, only by the integuments and the rectus femoris muscle, which was uninjured—the lateral, crucial and posterior ligaments were torn, the latter from its attachment to the back of the external condyle of the femur, which was rough for about the size of a shilling, as well as the corresponding part of the internal condyle, from which the inner head of the gastrocnemius muscle appeared to be torn—the synovial membrane was thickened and pulpy, and stained from the extravasated blood. There was an opening in the popliteal artery, more than half an inch long by nearly a third of an inch broad—the edges of the opening were thin and uneven. The vein and nerve were uninjured.

### II. Incision into the Knee-Joint.—Recovery.

Patrick Giltename, ætat 30 years, a very tall muscular man, was admitted 14th July 1834. He stated that, about six months previously, he fell while running quickly, and that his right knee was violently turned in, which caused great pain and swelling immediately and obliged him to remain in bed for some days, during which time he used warm stupes. Subsequently a medical man recommended him to rub the joint with a liniment. He recovered so far as to be able to walk about—the knee however continuing enlarged—until a week since, when he was suddenly attacked with severe pain and swelling of the same knee without any evident cause—it was again stuped but without relief. On admission the inflammation was considerable and principally on the inner side of the point where there was some redness of the skin, but little increase of pain on pressure—the patella was elevated evidently by effusion into the joint. Leeches were applied and the usual antiphlogistic treatment adopted for some days with much benefit. Another surgeon saw the patient on the 21st, who thought that the swelling was caused by effusion into, or thickening of the coverings of the joint, and was anxious to make an incision through the integuments to satisfy himself—he however continued the incision for nearly an inch in length into the joint, and gave exit to a considerable quantity of turbid synovia, with flakes of coagulable lymph. The incision was closed by adhesive plaster. During the five or six following days, synovia and lymph escaped from the joint in small quantities, but there was no pain or inflammation. On the 30th July the report states that the incision was completely united, but the joint was still longer than the corresponding one of the other limb. He was discharged cured on the 18th of the next month, and has continued well since.

### III. Wound of the Knee-joint—Recovery.

Pat. Conners, aged 18 years, admitted 14th July, 1836. A servant states that, while employed cleaning knives three weeks ago, one of them fell, and the point entered his left knee-joint on the inner side of the patella, inflicting a wound about half an inch in length—there was some trifling bleeding at the time—he bandaged it up, paid little attention to it, and continued to work for five or six days, when, in consequence of the great increase of pain and swelling, he was obliged to give over, and was recommended to apply poultices to the wound. He was at this time admitted to an hospital, where he remained twelve days—he became dissatisfied, and caused himself to be removed to Barrington's Hospital.

On admission, there was considerable swelling of the entire joint—great heat and tenderness—and pain much increased on the slightest attempt to move the limb. The wound was ununited and covered by fungous granulations. The limb was placed on a straight splint—leeches and cold evaporating lotions frequently used—by which means the inflammation was subdued in a few days: the joint however continued enlarged about the wound, from which some purulent matter escaped for four days, when it ceased to flow—it appeared to come from the neighbourhood of the wound—and for ten days afterwards a small quantity of synovia escaped at each dressing, diminishing in amount daily, the wound continuing fungous; it however soon healed up, when the splint was removed, as the joint was stiff, and still a little enlarged. Blisters were now applied, and the blistered surface dressed with mercurial ointment. Frictions with stimulating liniments were subsequently used, and he left the hospital on the 21st of the next month.

I again saw this young man in the month of December following, at which time he could walk pretty well without assistance, had no uneasiness in the knee; it was however still somewhat stiff, and he could not straighten it perfectly, on account of the contraction of the hamstring muscles.

### IV. Wound of the Knee-joint.—Death.

This case was one of a lacerated wound from the explosion of gunpowder. Suppuration ensued, after some bad treatment on the part of a quack, and on the 24th day the patient died with hectic symptoms. He obstinately refused amputation throughout.

### V. Wound of the Knee-joint—Fatal Hæmorrhage from the Popliteal Artery.

James Connors, 18 years old. I saw this man for the first time Jan. 2nd, 1838. It appears, that on the 24th of last December he was leading a horse, when, by some accident, he was thrown down and the wheel of the cart passed over his right knee, inflicting a severe lacerated wound nearly two inches long, which opened the joint. Symptoms of inflammation quickly set in, with discharge from the joint, at first of synovia, but which soon became purulent. When I examined him, I found a very large abscess over the head of the tibia, which I opened. The lacerated wound was fungous; there was not much swelling of the joint, but it was very painful, and there was great constitutional disturbance. Fistulous openings subsequently formed at different parts of the joint, which discharged considerable quantities of pus, accompanied by the usual symptoms of hectic.

Jan. 12th. The discharge from the original wound, as well as from the openings in the joint, has nearly ceased.

Jan. 19th. His appetite and general health has improved—there is not any discharge from the joint, but it appears weak, as if the ligaments were destroyed. As I had some expectation at this period that anchylosis of the joint might take place, a splint was placed behind the knee, and bandages applied—the lacerated wound however still presented a fungous appearance.

Jan. 22d. While the limb was being dressed this day in my absence a profuse arterial hæmorrhage suddenly took place from all the openings about the knee;—it was arrested as soon as possible by the application of a tourniquet. On my return to town, I found the patient very weak and restless, the limb cold and swollen from the tourniquet.

The patient having consented to amputation, I summoned the other surgeons of the hospital, and removed the limb. A little bleeding occurred in the evening, and the stump was re-opened—a large bed-sore formed—it sloughed—and, on the 12th of February, the patient died.

On examining the amputated limb, I found the synovial membrane in most places destroyed—a large cavity filled with blood extended three or four inches along the back of the femur, between that bone and the muscles—there was scarcely any trace of the posterior ligament of the joint—the entire of the cartilage covering the articulating surfaces of the condyles of the femur and of the head of the tibia was removed, as well as the intercuticular cartilage. The crucial ligaments were apparently uninjured, and the posterior part of each of the condyles of the femur was carious for about the size of a shilling, particularly the outer condyle. There was a small opening about the fourth of an inch in length on the anterior surface of the popliteal artery, exactly opposite the carious spot on the back of the outer condyle, with which it appeared in contact, and from which the hæmorrhage had evidently come.

---

### St. Bartholomew's Hospital.

### 1. Clinical Remarks by Mr. Lawrence on the best Mode of treating Encysted Tumors of the Eyelids, containing Hair.

A young child was taken to Mr. Lawrence with a tumor in the neighbourhood of the external canthus of the eye. It was about the size of a horse-bean, forming a colourless elevation, with the integuments covering it loosely, so that they could be pinched up into a fold over the swelling, which was more fixed below. It was placed immediately behind, and a little above the junction of the lids; and was said, by the mother, to have been there from the time of birth. On performing the operation, the tumor was found covered by the orbicularis palpebrarum, and closely adherent to the external angular process of the frontal bone. The following practical remarks of Mr. Lawrence are worth noticing.

"Such tumors are not unfrequent in infants and young children, occupying the situation just described. I believe them to be congenital; at least the statement of the mother generally leads to this inference. Sometimes they are stationary: in that case, if the swelling is small, there is no necessity for operation, as it is perfectly indolent. I am acquainted with a gentleman who has had through life a rather larger growth of this kind: it causes an unnatural fulness near the extreme angle of the eye, not amounting to deformity, and has never been attended with the slightest uneasiness. The affection, as in the case just described, is a cyst containing fat, which is sometimes of oily consistence, sometimes firmer. I have always found short hairs mixed with it in various proportions. Is this admixture of hairs, which resemble those of the eyebrow in length, to be regarded as an exemplification of the principle so frequently observed in adventitious structures, viz. that they resemble in nature that of the textures in which they grow, or that of parts in their immediate vicinity? It is placed under the orbicularis, and adheres more or less firmly to the bone. You must bear in mind the two latter circumstances in operating; make a larger incision than the size of the swelling would seem to require; and pay especial attention to the complete removal of the cyst from the bone.

If a portion of the cyst is left, the wound will not close, and such an occurrence is very annoying to the patient, and considered discreditable to the operator. I saw a young lady, in whom such a tumor as those I have described had been removed from the root of the nose, at the interval between the two eyebrows. She was a handsome person, and had submitted to the operation for the removal of what she deemed a blemish, though it must have been very slight, as the tumor was inconsiderable. She was much worse off after the operation than before; for the wound did not heal, at least it sometimes scabbed over, and sometimes discharged. A probe introduced into the opening went down apparently to the bone. Having learnt the nature of the swelling, and that the operator had experienced unexpected difficulty in separating it from the bone, I concluded that a bit of the cyst had been left behind, and proposed an incision to ascertain that point, which was readily consented to. I found, closely adhering to the frontal bone, a small strip of the cyst, conspicuous by its white glistening surface, and having a few short hairs on it: this was easily removed, and a firm cicatrix was soon secured.

I once saw a small growth of similar nature, but with an external aperture large enough to admit an ordinary dressing probe, on the bridge of the nose. It was congenital; and the opening sometimes produced a kind of greasy discharge. A probe passed in about a third of an inch. Various applications had been made to the part ineffectually. I slit it open, and found a smooth shining membrane with small hairs upon it, almost imbedded in the ossa nasi. It was difficult to dissect it out completely; but the removal was accomplished, and the part healed soundly."

### II. Treatment of Strumous Ophthalmia.

Mr. Lawrence proceeds on the principle of counter-irritation—not a very new one certainly. His mode of using it is thus set forth.

"In these strumous subjects the occurrence of disease in a new part relieves that which was previously suffering. In imitation of this natural process, the tartar-emetic ointment was rubbed on the nape in both patients, so as to produce and maintain a considerable irritation; the sulphate of quinine was administered internally; the bowels were regulated by small doses of rhubarb; animal diet was allowed, when the appetite indicated the necessity for it; and the suffering organs were occasionally fomented with tepid water. Under this treatment both cases have recovered: the children can now open their eyes and bear the light as well as any body, and we find that no unfavourable change has occurred in the affected parts. The patients are at the same time improved in general health. In one of them, the pustular eruption caused by the ointment spread over the body, with considerable but temporary constitutional disturbance."

### Guy's Hospital.

### I. Dr. Addison on Electricity in certain Convulsive and Spasmodic Diseases.*

Dr. Addison was led to employ electricity in these cases, first, because other means were deplorably unsuccessful; and, secondly, because he had, by means of Mr. Golding Bird's assistance, an opportunity of employing it very effectually.

The mode of application is thus detailed by the electrician to the hospital, Mr. Frederick Bird.

"In the following cases, the form of electricity employed, was, with one exception, that elicited by means of the common electrical machine; being made use of either by taking sparks, in the course of the spine; or in the form of shocks, passed through the pelvis.

In the former case, the patient was seated on an insulated stool, and a metallic connexion made between the prime conductor of the machine and the body of the patient: a brass ball, furnished with a wire or chain, in connexion with the earth, was then passed upwards and downwards, in the direction of the spine, at a distance of about an inch from the surface. The machine being at this time excited, the patient became charged, and the electricity continued to pass off, accompanied by sparks, to the brass ball, and thence escaping, through the medium of the wire or chain, to the earth; in this manner a rapid succession of sparks could be maintained; and which, in the present instances, was continued until an eruption followed, which assumed very much the appearance of lichen urticatus; the time necessary for its production varying, in different patients, from five to ten minutes.

For the purpose of passing the shocks, the following method was had recourse to. A large-sized Leyden jar was so placed, that a communication was established between its inside coating and the prime conductor: a 'Lane's electrometer,' was then fixed into one end of the conductor, so as to admit of the insulated ball of the former instrument being either in contact with, or at any required distance from, the latter: a chain was placed in contact with the outside coating of the jar, and another was attached to the ball of the electrometer; the ends of both of which were furnished with directors, for convenience of application.

One of the directors was then held upon the symphysis pubis, whilst the other was placed upon the sacrum;† by which means the electric current, in performing its circuit, was made to pass through the pelvis. The ball of the electrometer being then placed at a certain distance (generally $\frac{3}{8}$ths of an inch) from the prime conductor, motion was given to the machine, and the charging of the jar commenced; and upon a sufficient quantity of electricity being accumulated to enable its discharge to take place by means of the electrometer, the shock was felt. By adopting the use of an electrometer of this kind, the violence of the shocks is made to depend upon the distance of its insulated ball from the conductor of the machine, and not upon the capacity of the jar: hence, it is only necessary to place the ball at a greater or less distance from the conductor, in order to proportion the intensity of the discharge to the nature of the disease, or powers of the patient.

In one case, that of Jessie Wick, the magnetic-electrical machine was made

---

* Guy's Hospital Reports, No. V.

† "There is a female in attendance, for the purpose of adjusting that part of the apparatus more immediately connected with the person of the patient."

use of, the patient's strength not being sufficient to admit of the ordinary and more powerful form of electricity. The larger helix having been adjusted to the machine, one of the two conducting wires, furnished with a brass disk, was placed over the cervical portion of the spine; whilst the remaining wire, which was also provided with a disk, was fixed over the lumbar vertebræ: the helix being then slowly revolved, a succession of shocks was obtained, which were thus made to traverse the course of the spinal column."

Seven cases, taken from the hospital-books are detailed.

*Case* 1. Jessie Wick, aged 17, admitted May 14th, 1837. She had had irregular menstruation, and two months previously, being frightened while menstruating, she fell into the following condition:—She is utterly unable to remain in a state of rest for a moment: her limbs, especially the upper extremities, are violently agitated: the mouth is from time to time ludicrously distorted. The most unvarying motion is, a rolling of her clenched hands quickly round each other, with a thrusting forward of the right in a very systematic manner, it occurring after every third revolution. Deglutition and speech but slightly affected. Occasional pain in the head, back and loins, and under the left mamma: palpitation of the heart, but no abnormal sound. Her spirits are good, but she is fatigued from the continued action of her muscles.

Purgatives—creosote—hydrocyanic acid—ultimately iron, produced such amendment, that she left the hospital for Ramsgate. But, in October, she returned worse than ever. She had had, in the interim, epileptic fits. When re-admitted, she had a foolish imbecile stare, the face dull, she appeared to be almost regardless of surrounding objects, articulation was lost, and she made no attempt, even by signs, to express her feelings. The twitchings of the upper extremities, mouth, and eyes, were less: the inferior extremities appeared paralytic, at least she made no attempt to move them, nor was she able to stand; her bowels were said to be regular; there had been no return of the catamenia. She was immediately put upon ℈j. doses of carbonate of iron, and used the shower-bath; and a blister was applied to the spine, along which there seemed to be pain on pressure. The bowels becoming costive, drastic purgatives were again necessary, and croton-oil was given. On the 20th of October the sulphate was substituted for the carbonate of iron; but under this treatment no amendment was produced. After one of her fits, she lay perfectly comatose, scarcely seeming to breathe; and it was not till repeated assafœtida injections had brought away large quantities of fæcal matter, deeply tinged with iron, and stimulating remedies had been used, that her consciousness, and with it articulation, were regained. It would be useless to enumerate the additional symptoms—severer ones of the same description.

As a last resource, Dr. Addison ordered electricity. Her strength not allowing of the severer application, electro-magnetism was commenced. It caused continued spasm of the flexor-muscles of the arm; so that, till the current was discontinued, she could not relax her grasp of the brass handles. This was commenced on the 20th of April; and on the 10th of the following month she was so far improved, that she could use her needle with tolerable precision: her general health improved; and the fits became slighter, though as frequent as before. Sparks were now drawn from the spine every other day; each exhibition continuing till a vivid eruption was produced. Her improvement was most marked: at the end of a week she was able to walk across the room without assistance: her countenance gradually became less anxious, and the fits declined in frequency.

*June* 1. Twelve shocks, through the pelvis, every other day, were ordered. The first administration, at the distance of three-eighths of an inch from the conductor, was followed by severe abdominal and pelvic pains, the immediate precursors of the catamenia. The secretion continued for four hours. Shocks to be discontinued.

*July* 3. Improvement uninterrupted: occasional twitchings are the only indications of chorea. The catamenia have not appeared this month.

A second exhibition of the shocks again occasioned the development of the catamenial function: in six hours it became arrested; after which, she vomited a small quantity of blood.

*July* 15. She left the hospital entirely free from chorea; though still subject to fits of diminished force and frequency.

*Case* 2. This was one of chorea. Two doses of calomel and rhubarb, and electricity for three weeks, cured it.

*Case* 3. Chorea. Cured in less than a month. The patient had had two attacks before, cured by other remedies.

*Case* 4. *Hysterical Paralysis.* A girl, of 16, had had her catamenia suspended for six months. Ten days before admission, the face became numb on the left side, the sight of the left eye became dim, and there was a slight pain in the globe. On the third day there was perfect amaurosis and ptosis. At the time of admission, there was numbness, coldness, and deficient muscular power over the whole of the left side, including the lining membrane of the mouth, nostril, and conjunctiva: in the latter she perceived a burning sensation, but could not appreciate a touch: the pupil was contracted, and not at all obedient to light: she could not raise the upper eyelid. She complained of pain in the head, and giddiness. Bowels torpid.

C. C. Nuchæ.—Emp. Lyttæ postea.

Mist. Magnes. c̄ Magnes. Sulph. et Tinct. Jalapæ, ʒj. t. d.

This treatment was continued for some time, without the slightest benefit.

*April* 24. Dr. Addison ordered electricity, in the shape of sparks, down the spinal column. On the same evening she could bend her fingers; and the 27th recovered, to a certain extent, power over the muscles of the arm. The third application, on the 28th, restored vision, and the power of elevating the upper eyelid. In June, electrical sparks were drawn from the left eyelid, and shocks passed through the uterus. She could walk, but the eye was amaurotic, and in the condition in which it was on admission. The catamenia returned on the 11th of June, and she left the hospital.

*Case* 5. Convulsions, especially on the right side—epilepsy—headache—and many severe symptoms, apparently indicative of organic lesion. Electricity seems to have done *some* good.

*Case* 6. Severe chorea—cured in less than a month by electricity.

*Case* 7. Chorea—cured by electricity. It returned, and was cured by sulphate of zinc.

### II. Dr. Ashwell on the Speculum in Diseases of the Uterus.

We extract these observations by an experienced obstetrical physician, on the use of an instrument which is now attracting much attention—the speculum. They seem to us to be very fair.

"While the touch enables us to recognise structural changes in the bulk, firmness, and sensibility of these parts, the sight rectifies and perfects an erroneous or incomplete opinion, by shewing the *nature and limits of ulceration, excoriation or eruption*, the *appearance* of the cervix and vagina in various stages of disease, and the *colour* and *consistency* of the accompanying discharges.

The best and most easily used speculum is made of tin, with an inner, highly-polished surface. There need be no division in the cylinder, and the complicated screw is not required. I have, for hospital use, a series of these conical tubes, of various sizes; and the previous introduction of the finger into the vagina enables me to select the right-sized speculum. The length of the tube should be from five to seven inches, and it may or may not have a handle: on the whole, it is, perhaps, more readily used without one. The strong light of the sun is the best for these examinations, but a candle is an excellent substitute.

The rules prescribed for the introduction of obstetric instruments into the vagina will serve here. The labia being widely separated, the speculum is to be carefully and slowly passed, backwards and downwards, towards the point of the coccyx. The principal obstacle is at the entrance of the vagina; for when the resistance of its sphincter is once overcome, the speculum will easily traverse the rest of the canal. Care must be taken that the transverse portion of mucous membrane, placed posteriorly, called the fourchette, is not stretched and carried forward by the instrument, as great pain and difficulty in the introduction will be the result.

The position of the neck is occasionally changed, being placed more forward or posteriorly than natural. To obviate this difficulty, and to bring the cervix within the end of the tube, the speculum must be elevated or depressed. Sometimes, from spasmodic contraction, induced by the passing of the cylinder, a fold of the mucous membrane of the vagina is forced into the aperture of the speculum, and may be mistaken for the cervix: the least movement, however, of the instrument will cause the slipping away of the portion thus placed; and the recognition of the neck, which is glandular, smooth, and without rugæ, and paler than the vagina, is not difficult.

The whole circumference of a very large cervix cannot be examined at once: the position of the speculum requires attention, and if the parts are not morbidly sensitive, the instrument is easily and safely turned in the vagina: this caution is important; as very lately I overlooked a rather large ulcer on the inferior and posterior surface of the neck, from a neglect of it."

"Valuable as is the speculum, its use has been indiscriminately and unnecessarily urged. In slight cases of uterine irritation and leucorrhœa, its employment is prejudicial; while, in leucorrhœal discharges of long standing, and in menorrhagia of months' and years' continuance, its introduction cannot be too strongly recommended: for it must never be forgotten, that these maladies rarely exist long, without more or less of organic change. If there be a suspicion of structural mischief about the lower part of the uterus, there ought to be no delay, not only in touching, but in seeing the seat of the suspected disease.

There are circumstances which entirely forbid the employment of the speculum. In very young and very old persons, its introduction is difficult, and sometimes altogether impossible, without laceration. The hymen in the young, and the great shrinking and contraction of the vagina in aged women, present obstacles so serious, that the use of the speculum ought to be given up, unless the necessity be extremely urgent. I have several times found membranous bands stretching across the vagina, and contractions of its caliber from cicatrices, which would have entirely impeded the passage of the instrument. There was lately an out-patient of mine at Guy's, whose vagina was so funnel-shaped at its upper part, as to preclude my touching the os or cervix, except by a probe, introduced through the minute aperture at the apex of the funnel by which the catamenia escaped from the uterus. Steatomatous tumors occupying the walls of the vagina, ovarian growths in the recto-vaginal septum, polypi, deep ulcerations of the vagina or neck of the uterus, large cauliflower excrescences, or bleeding fungi, all contra-indicate the use of the speculum. When the neck is inflamed, or much congested, or where the vagina is excessively sensitive, the introduction of the speculum should be deferred, till these various morbid conditions are ameliorated."

The speculum has been over-rated, but it is a valuable means of diagnosis. It is a pity that the gross indecency of it, makes it a derniere instead of a premiere resource.

